# Diverse mechanisms associated with cyhalofop-butyl resistance in Chinese sprangletop (*Leptochloa chinensis* (L.) Nees): Characterization of target-site mutations and metabolic resistance-related genes in two resistant populations

**DOI:** 10.3389/fpls.2022.990085

**Published:** 2022-11-28

**Authors:** Yi Zhang, Liping Chen, Wen Song, Tao Cang, Mingfei Xu, Changxing Wu

**Affiliations:** Institute of Agro-product Safety and Nutrition, Zhejiang Academy of Agricultural Sciences, Hangzhou, Zhejiang, China

**Keywords:** *Leptochloa chinensis*, cyhalofop-butyl, herbicide resistance, target-based resistance, metabolic resistance, RNA sequencing

## Abstract

Resistance of Chinese sprangletop (*Leptochloa chinensis* (L.) Nees) to the herbicide cyhalofop-butyl has recently become a severe problem in rice cultivation. However, the molecular mechanisms of target-site resistance (TSR) in cyhalofop-butyl*-*resistant *L. chinensis* as well as the underlying non-target-site resistance (NTSR) have not yet been well-characterized. This study aimed to investigate cyhalofop-butyl resistance mechanisms using one susceptible population (LC-S) and two resistant populations (LC-1701 and LC-1704) of *L. chinensis.* We analyzed two gene copies encoding the entire carboxyltransferase (CT) domain of chloroplastic acetyl-CoA carboxylase (ACCase) from each population. Two non-synonymous substitutions were detected in the resistant *L. chinensis* populations (Trp^2027^-Cys in the *ACCase*1 of LC-1701 and Leu^1818^-Phe in the *ACCase*2 of LC-1704), which were absent in LC-S. As Trp^2027^-Cys confers resistance to ACCase-inhibiting herbicides, the potential relationship between the novel Leu^1818^-Phe mutation and cyhalofop-butyl resistance in LC-1704 was further explored by single-nucleotide polymorphism (SNP) detection. Metabolic inhibition assays indicated that cytochrome P450 monooxygenases (P450s) and glutathione *S*-transferases (GSTs) contributed to cyhalofop-butyl resistance in specific resistant populations. RNA sequencing showed that the P450 genes *CYP71Z18*, *CYP71C4*, *CYP71C1*, *CYP81Q32*, and *CYP76B6* and the GST genes *GSTF11*, *GSTF1*, and *GSTU6* were upregulated in at least one resistant population, which indicated their putative roles in cyhalofop-butyl resistance of *L. chinensis*. Correlation analyses revealed that the constitutive or inducible expression patterns of *CYP71C4*, *CYP71C1*, *GSTF1*, and *GSTU6* in *L. chinensis* were strongly associated with the resistant phenotype. For this reason, attention should be directed towards these genes to elucidate metabolic resistance to cyhalofop-butyl in *L. chinensis*. The findings of this study improve the understanding of mechanisms responsible for resistance to ACCase-inhibiting herbicides in grass-weed species at the molecular level, thus aiding in the development of weed management strategies that delay the emergence of resistance to this class of pest control products.

## Introduction

Chinese sprangletop (*Leptochloa chinensis* (L.) Nees), a tetraploid (2*n*=4*x*=40) belonging to the Chloridoideae subfamily of the Poaceae (grass) family, is a pernicious weed affecting rice production in Asia (Wang et al., 2022b). It competes with rice crops for light, water, and soil nutrients (Oerke and Dehne, 2004). Acetyl-CoA carboxylase (ACCase) -inhibiting herbicides were developed in the mid-1970s, which block fatty acid biosynthesis in sensitive grassy weeds (Secor et al., 1989). These highly effective herbicides have been widely used to control various weed species. Cyhalofop-butyl is an ACCase-inhibiting herbicide commonly used to control *L. chinensis* in paddy fields (Ruiz-Santaella et al., 2006). However, repeated use of cyhalofop-butyl has exerted selection pressure on weeds and caused the emergence of resistant *L. chinensis* populations in several countries including China, South Korea, Thailand, and Malaysia (Rahman et al., 2011; Phongphitak et al., 2014; Heap, 2022).

Rice farmers in China typically respond to herbicide resistance by increasing application rates. However, this practice, in addition to eluding the principles of integrated weed management, increases the risk of hazardous substance release into the environment, and harms non-target organisms. Hence, herbicide resistance mechanisms must be elucidated to design more effective weed control strategies, and by extension, help prevent or delay the emergence of herbicide resistance.

Resistance to ACCase inhibitors may be due to different mechanisms, target-site (TS) or non-target-site (NTS) mediated. In TS resistance (TSR), the ACCase binding site is altered by non-synonymous mutations in the carboxyltransferase (CT) domain. There is empirical evidence that at least 17 mutations in seven codons may contribute to TSR in various weed species. These mutations include Ile^1781^-Val/Leu/Thr, Trp^1999^-Cys/Leu/Ser, Trp^2027^-Cys/Ser/Leu, Ile^2041^-Asn/Val/Thr, Asp^2078^-Gly/Glu, Cys^2088^-Arg, and Gly^2096^-Ala/Ser (Kaundun, 2014; Guo et al., 2017; Fang et al., 2020; Peng et al., 2020; Zhao et al., 2022). In NTS resistance (NTSR), the amount of the herbicide reaching the target site is reduced by increased excretion, sequestration or metabolism, and decreased herbicide uptake and translocation (Gaines et al., 2020). Metabolic resistance has been associated with cytochrome P450 monooxygenases (P450s) or glutathione *S*-transferases (GSTs) upregulation in weed species such as *Lolium* spp. (Busi et al., 2011; Cummins et al., 2013), *Echinochloa phyllopogon* (Yun et al., 2005; Iwakami et al., 2019), *Poa annua* (Wang et al., 2013), *Alopecurus aequalis* (Wang et al., 2013), *Alopecurus myosuroides* (Brazier et al., 2002; Cummins et al., 2013), and *Avena sterilis* (Maneechote et al., 1997). However, the molecular mechanisms underlying metabolic resistance are complex, diverse, and therefore, unclear (Gaines et al., 2020). Additionally, multiple herbicide resistance mechanisms may occur within the same species, population, or individual (Gaines et al., 2020). For instance, TSR and NTSR mechanisms co-occur in pinoxaden-resistant *Lolium multiflorum* populations (Kaundun et al., 2013a). Moreover, the combined effects of an Ile^1781^-Leu mutation and other mechanisms were suggested to be responsible for ACCase-inhibiting herbicide resistance in an *A. myosuroides* population (Kaundun et al., 2013b).

Molecular research on cyhalofop-butyl resistance in *L. chinensis* has focused mainly on TSR mechanisms with different ACCase mutations between populations. Trp^2027^-Cys substitution might explain cyhalofop-butyl resistance in the ZHYH *L. chinensis* population (Yu et al., 2017). The same mutation was also detected in a subsequent study on the SJ-8 *L. chinensis* population (Deng et al., 2019). These authors also reported the Ile^1781^-Leu and Trp^1999^-Leu substitutions in various cyhalofop-butyl-resistant populations. The mutations Trp^2027^-Ser and Ile^2041^-Asn were found in the resistant LC-18011 and LC-17009 populations, respectively (Peng et al., 2020), with the former mutation being detected in the resistant CM9-1 population as well (Yuan et al., 2021). The novel Trp^2027^-Leu mutation was observed in the resistant HFFD3 population (Zhao et al., 2022). Metabolic resistance may nonetheless emerge in cyhalofop-butyl-resistant *L. chinensis* (Deng et al., 2021). Upregulation of P450- and GST-associated metabolism might contribute to cyhalofop-butyl resistance in this species (Zhao et al, 2022). A whole-transcriptome analysis of *L. chinensis* indicated six candidate genes associated with cyhalofop-butyl NTSR in *L. chinensis* (Chen et al., 2021). However, the exact functions of these genes were not clarified.

The mechanisms involved in cyhalofop-butyl resistance may differ among *L. chinensis* populations. Several alterations in ACCase are apparently associated with cyhalofop-butyl resistance. Nevertheless, as the current TSR studies for cyhalofop-butyl-resistant *L. chinensis* mainly concentrate on detecting resistance-conferring mutation sites in the partial ACCase CT domain, the spectrum of mutations in the entire ACCase CT domain has not been fully characterized to date. Consequently, the molecular mechanisms of metabolic resistance to cyhalofop-butyl in *L. chinensis* have received relatively little attention. In the present study, we investigated the molecular mechanisms involving TSR and metabolic resistance in two cyhalofop-butyl-resistant *L. chinensis* populations. The key objectives of this study were to:

• clone the gene encoding the entire ACCase CT domain in *L. chinensis* to identify putative mutations associated with cyhalofop-butyl resistance;
• examine the effects of various metabolic inhibitors on cyhalofop-butyl toxicity in susceptible and resistant *L. chinensis* populations;
• perform RNA sequencing (RNA-Seq) to validate candidate P450 and GST genes responsible for metabolic resistance to cyhalofop-butyl in *L. chinensis*.

## Materials and methods

### Chemicals

Nine ACCase-inhibiting herbicides were used for the bioassays and the information on each is summarized in **Table 1**. Piperonyl butoxide (PBO, 95%) and cyanuric chloride (99%) were purchased from Macklin Biochemical Co. Ltd. (Shanghai, China) and used in the metabolic inhibitor effect assays. All other chemicals were of analytical quality and obtained from commercial suppliers.

**Table 1:** ACCase-inhibiting herbicides applied for the bioassay.

Group^a^	Herbicide	Formulation^b^	Company
APP	Cyhalofop-butyl	100 g L^-1^ EC	Dow AgroSciences, Beijing, China
	Metamifop	10% EC	FMC, Jiangsu, China
	Fenoxaprop-*P*-ethyl	69 g L^-1^ EW	Bayer, Beijing, China
	Haloxyfop-R-methyl	108 g L^-1^ EC	Flag Chemical, Jiangsu, China
	Quizalofop-*P*-ethyl	5% EC	Zhongnonglihua, Tianjin, China
	Clodinafop-propargyl	15% ME	Meicheng, Anhui, China
	Fluzifop-*P*-butyl	15% EC	Shiyuanjinniu, Zhejiang, China
CHD	Sethoxydim	12.5% EC	Cynda, Shandong, China
DEN	Pinoxaden	5% EC	Syngenta, Shanghai, China

a APP, aryloxyphenoxypropionate; CHD, cyclohexanedione; DEN, phenylpyrazoline.

b EC, emulsifiable concentrate; EW, emulsion in water; ME, microemulsion.

### Plant material

The three *L. chinensis* populations were sampled in Zhejiang Province, China. Seeds of the susceptible LC-S population were collected in 2016 from non-cultivated land without any prior herbicide treatment; two resistant populations (LC-1701 and LC-1704) which had survived previous cyhalofop-butyl application in rice fields were sampled in 2017.

### Herbicide sensitivity bioassays

Whole-plant pot experiments were performed to determine the sensitivities of the three *L. chinensis* populations to cyhalofop-butyl and eight other ACCase-inhibiting herbicides (Wang et al., 2013). Approximately 30 seeds were sown in plastic pots (9 cm diameter) containing loam soil. The pots were then placed in a climate chamber under a 25°C/20°C, day/night temperature regimen and a 14 h/10 h, light/dark cycle. The seedlings were watered as required. When most of the *L. chinensis* seedlings reached the two- to three-leaf stage, they were thinned to ten plants per pot. At the three- to four-leaf stage, they were sprayed with ACCase-inhibiting herbicides according to the application method described by Zhang et al. (2021). The doses of each herbicide applied to the susceptible and resistant *L. chinensis* populations are determined on the basis of preliminary experimental results and listed in **Table S1**. The control plants were sprayed with pure water only. Each treatment was performed in triplicates, and the experiment was conducted twice. Shoot fresh weights were recorded at 21 days after treatment (DAT).

### ACCase CT domain cloning and sequence analysis

The shoots of ten individuals per population were pooled and used for total RNA extraction with the RNAprep Pure Plant Kit (Tiangen, Beijing, China) according to the manufacturer’s instructions. RNA quantity and integrity were determined by Nanodrop spectrophotometry (Thermo Fisher Scientific, Waltham, MA, USA) and gel electrophoresis, respectively. One microgram total RNA per sample was reverse-transcribed with a PrimeScript™ RT Reagent Kit with gDNA Eraser (Takara Biotechnology, Dalian, China) according to the manufacturer’s instructions. The fragment encoding the entire ACCase CT domain was amplified with the primers LC-CT-F (5’-AGTAGAAATAACCAATCCTATGTC-3’) and LC-CT-R (5’-AGTGCTTCCTGTGTCTACTTG-3’). The primers were designed based on the conserved *ACCase* regions in closely related species including *Echinochloa crus-galli* (NCBI accession No. HQ395758), *A. myosuroides* (NCBI accession No. AJ310767), *Triticum aestivum* (NCBI accession No. AF029895), and *Zea mays* (NCBI accession No. U19183). The thermocycling program was 98°C for 2 min followed by 35 cycles of 98°C for 10 s, 52°C for 15 s, and 72°C for 90 s and a final extension at 72°C for 5 min. The purified PCR products were cloned into a pClone007 Blunt Simple Vector (Tsingke Biotechnology, Beijing, China). Fifteen positive clones per sample were selected and sequenced. The nucleotide and amino acid sequences of the cDNA fragment were aligned with DNAman v. 9.0 (Lynnon Biosoft, San Ramon, CA, USA). Using the amino acid sequences of the ACCase CT domains from other plant species, a phylogenetic tree was constructed using MEGA X’s neighbor-joining method (Saitou and Nei, 1987).

### Single-nucleotide polymorphisms of the partial CT domains in various *L. chinensis* populations

Thirty individuals per *L. chinensis* population were collected and used for SNP determination. Total RNA extraction and first-strand cDNA synthesis were conducted as previously described. Based on the sequence analyses of *ACCase* in the susceptible and resistant populations, the LC-F1 (5’-TATACATGGAAGTGCTGCTA-3’) and LC-R1 (5’-TCCTCCACGTAGCTCTCC-3’) primers were used to amplify the 877-bp fragment bearing the putative polymorphisms associated with cyhalofop-butyl resistance. The thermocycling program was 98°C for 2 min followed by 35 cycles of 98°C for 10 s, 52°C for 15 s, and 72°C for 30 s, and a final extension at 72°C for 5 min. The PCR products were directly sequenced. According to the results of sequence analysis, alleles at codon position 1818 and 2027 were visualized with a Chromas Chromatogram Viewer (Technelysium Pty. Ltd., South Brisbane, QLD, Australia). Allele frequencies were recorded for each polymorphic site and in all tested individuals.

Another SNP detection was conducted on the LC-1704 population to determine the potential contribution of the novel Leu^1818^-Phe mutation to cyhalofop-butyl resistance. A total of 883 LC-1704 individuals at the three- to four-leaf stage were exposed to 4,413 g a.i. ha^-1^ cyhalofop-butyl which corresponds to its 90% effective dose (ED_90_) value. Twenty-one individuals had survived at 21 DAT; these plants were collected and designated as the LC-1704H group, which was considered to be highly resistant to cyhalofop-butyl. Genomic DNA from each LC-1704H individual was extracted with a Plant Genomic DNA Extraction Kit (Tsingke). The primers LC-F1 and LC-R2 (5’-GGATAGCTGAACGAGGAT-3’) were used to amplify the 435-bp fragment containing the codon position 1818. The thermocycling program was 98°C for 2 min followed by 35 cycles of 98°C for 10 s, 54°C for 15 s, and 72°C for 15 s, and a final extension at 72°C for 5 min. The subsequent allele analysis at codon position 1818 was conducted as previously described.

### Homology modeling of the entire ACCase CT domain

The deduced amino acid sequence of the entire ACCase CT domain of *L. chinensis* was modeled on the Swiss-Model Homology Modeling Server (https://swissmodel.expasy.org/interactive ). The Chiron Server developed by the Dokholyan Laboratory (https://dokhlab.med.psu.edu/chiron/login.php ) and a structural analysis and verification server from UCLA (https://saves.mbi.ucla.edu ) were used to refine and validate the protein structure, respectively. The 3D structure of the entire ACCase CT domain of *L. chinensis* was visualized with PyMOL Viewer (https://pymol.org/2/).

### Metabolic inhibition assays

Metabolic inhibition was evaluated with PBO and cyanuric chloride which inhibit P450s and GSTs, respectively (Cummins et al., 2013; Wang et al., 2013). The metabolic inhibitors were dissolved in acetone, diluted in distilled water, and applied to plants of each *L. chinensis* population 1 h before cyhalofop-butyl treatment at a dose of 500 g a.i. ha^-1^. Based on a preliminary experiment, the doses of the inhibitors were experimentally selected as the highest doses that had no phytotoxic effects on their own. The subsequent treatments were performed in accordance with the previously described whole-plant pot experiments.

### RNA-Seq data analysis

The LC-S, LC-1701, and LC-1704 *L. chinensis* populations were subjected to RNA-Seq analysis. Plants at the three- to four-leaf stage were sprayed with cyhalofop-butyl at the recommended field application rate in China, namely, 105 g a.i. ha^-1^. Controls were sprayed with pure water. For both cyhalofop-butyl-treated and control groups, leaves were excised from ten individuals per population at 24 h after treatment and were rapidly frozen in liquid nitrogen. The cyhalofop-butyl-treated groups were designated as LC-S-T, LC-1701-T, and LC-1704-T while the controls were designated as LC-S-C, LC-1701-C, and LC-1704-C. There were three biological replicates per group. Hence, 18 samples were collected and stored at -80°C until RNA extraction.

Extraction, quality assessment, and quantity determination of total RNA from each of the 18 samples were performed as previously described. RNA quality was also evaluated with the RNA chip on an Agilent 2100 Nano device (Agilent Technologies, Santa Clara, CA, USA). The cDNA libraries were constructed for each sample and sequenced on an Illumina HiSeq 2500 platform (Illumina, San Diego, CA, USA). *De novo* transcriptome analysis was then performed. Clean data were produced by removing low-quality reads and adaptor sequences with fastp (https://github.com/OpenGene/fastp ). To generate the transcriptome reference sequence, clean data for the 18 samples were merged for assembly with Trinity (https://github.com/trinityrnaseq/trinityrnaseq ). TransRate (https://github.com/Blahah/transrate ) and CD-HIT (https://github.com/weizhongli/cdhit ) were used for quality filtering. BUSCO (https://gitlab.com/ezlab/busco/-/releases#5.2.1 ) was used to assess the reference transcriptome assemblies. Clean reads from each *L. chinensis* sample were separately mapped against the reference transcriptome. Genes and transcripts were functionally annotated with the Gene Ontology (GO; geneontology.org), Kyoto Encyclopedia of Genes and Genomes (KEGG; https://www.genome.jp/kegg/ ), Clusters of Orthologous Groups (COG; https://www.ncbi.nlm.nih.gov/research/cog-project/ ), non-redundant protein (NR; https://www.ncbi.nlm.nih.gov/refseq/about/nonredundantproteins/ ), Swiss-Prot (https://www.expasy.org/resources/uniprotkb-swiss-prot ), and Pfam (https://www.uniprot.org/database/DB-0073 ) databases.

The read counts per gene were normalized as transcripts per million reads. Gene expression abundances were calculated with RNA-Seq and expectation-maximization (Li and Dewey, 2011). DESeq2 (https://bioconductor.org/packages/release/bioc/html/DESeq2.html ) was used to identify differentially expressed genes (DEGs) between susceptible and resistant plants with or without cyhalofop-butyl treatment. Genes with │log_2_FC│ ≥ 1 and *P* < 0.05 were considered differentially expressed. The GO annotations were analyzed for functional DEG classification.

### Candidate detoxification enzymes associated with cyhalofop-butyl resistance in *L. chinensis*

To screen DEGs associated with enhanced herbicide detoxification, attention was directed towards those encoding P450s and GSTs because they were previously suggested to be implicated in metabolic resistance to herbicide. Gene expression was analyzed for LC-1701-C *vs*. LC-S-C, LC-1704-C *vs*. LC-S-C, LC-1701-T *vs*. LC-S-T, and LC-1704-T *vs*. LC-S-T. Since our metabolic inhibition assays showed that the detoxification enzymes regulating cyhalofop-butyl resistance differed between LC-1701 and LC-1704, the selection criteria for the P450 genes differed from those for the GST genes. Upregulated P450 genes in the LC-1701 or LC-1704 samples were considered as candidates. By contrast, only GST genes that were upregulated in the LC-1701 but not in the LC-1704 plants were selected for further validation.

The expression patterns of the candidate genes were validated by quantitative reverse-transcription polymerase chain reaction (qRT-PCR). *EF1α*, *eIF4a*, and *CAP* served as internal reference genes (Zhang et al., 2021). The primers used in qRT-PCR are listed in **Table S2**. The PCR was performed in a CFX96 Real-Time PCR System (Bio-Rad Laboratories, Hercules, CA, USA) with a TB Green Premix Ex Taq™ II Kit (Takara Biotechnology). The PCR program used was previously published (Zhang et al., 2021). Relative expression levels were calculated by the 2^-△△Ct^ method (Livak and Schmittgen, 2001). The preparation of *L. chinensis* samples used for qRT-PCR was consistent with that used for RNA-Seq. The experiment was independently conducted in triplicates using different RNA samples, including those prepared for RNA-Seq.

### Confirmation of candidate genes associated with cyhalofop-butyl resistance in *L. chinensis*

LC-1701 and LC-1704 plants at the three- to four-leaf stage were used to generate *L. chinensis* groups with various levels of resistance to cyhalofop-butyl. Based on the bioassay results, individuals were separately treated with cyhalofop-butyl at their respective 10% effective dose (ED_10_) and ED_90_ (For LC-1701, ED_10_ = 36 g a.i. ha^-1^ and ED_90_ = 967 g a.i. ha^-1^. For LC-1704, ED_10_ = 123 g a.i. ha^-1^ and ED_90_ = 4,413 g a.i. ha^-1^.). Approximately 250 individuals were used per treatment. Seven days after treatment, individuals that survived to ED_10_ dose and presented visible symptoms were collected and designated as LC-1701-ED_10_ and LC-1704-ED_10_. Plants that survived to ED_90_ dose at 21 DAT were designated as LC-1701-ED_90_ and LC-1704-ED_90_. F_1_ seeds from all four groups were separately collected and designated as LC-1701-ED_10_-F_1_, LC-1704-ED_10_-F_1_, LC-1701-ED_90_-F_1_, and LC-1704-ED_90_-F_1_. F_1_ progeny from LC-S, LC-1701, and LC-1704 were also collected and designated as LC-S-F_1_, LC-1701-F_1_, and LC-1704-F_1_, respectively.

To elucidate the associations between candidate detoxification enzyme genes and cyhalofop-butyl resistance in *L. chinensis*, bioassays were conducted on each of the seven F_1_ groups as previously described. The cyhalofop-butyl treatment doses were: 0, 1.8, 3.5, 7, 14, 28, and 56 g a.i. ha^-1^ for LC-S-F_1_ plants; 0, 18, 35, 70, 140, 281, 563, and 1 125 g a.i. ha^-1^ for the LC-1701-F_1_, LC-1701-ED_10_-F_1_, and LC-1701-ED_90_-F_1_ plants; and 0, 70, 140, 281, 563, 1 125, 2 250, and 4 500 g a.i. ha^-1^ for the LC-1704-F_1_, LC-1704-ED_10_-F_1_, and LC-1704-ED_90_-F_1_ plants. Based on the preliminary validation results for candidate gene transcriptional levels in the susceptible and resistant populations, *CYP71Z18*, *CYP71C4*, *CYP71C1*, *CYP81Q32*, *CYP76B6*, *GSTF11*, *GSTF1*, and *GSTU6* were selected for further expression validation. Given that the expression pattern of each gene differed between two resistant populations (**Table S3**), different groups of F_1_ progeny were used to determine the expression level for each gene (**Table S4**). The preparation of F_1_ samples (with or without cyhalofop-butyl treatment) used for further qRT-PCR was consistent with that of the *L. chinensis* samples used for RNA-Seq. The qRT-PCR program used was previously described.

### Data analyses

A probit analysis of the bioassays was performed in DPS v. 18.10 (Data Processing System, Hangzhou, China) to compute the 50% effective dose (ED_50_) value. Herbicide resistance ratios were calculated by dividing the herbicide ED_50_ for the corresponding resistant population by the herbicide ED_50_ for the susceptible population. The synergism ratio was calculated by dividing the ED_50_ for the herbicide alone by the ED_50_ for the herbicide combined with the corresponding metabolic inhibitor.

Differences in the gene expression levels between the susceptible and resistant populations were separately analyzed in each of the untreated and cyhalofop-butyl-treated groups using one-way ANOVA followed by the LSD test. Pairwise correlations were determined by Pearson’s correlation analysis. Statistical analyses were conducted in SPSS v. 20.0 (IBM Corp., Armonk, NY, USA). Statistical significance was indicated at *P* < 0.05.

## Results

### Resistance levels to cyhalofop-butyl and other ACCase-inhibiting herbicides in *L. chinensis*

Compared with the susceptible LC-S population, the LC-1701 and LC-1704 *L. chinensis* populations were highly resistant to cyhalofop-butyl (ED_50_ = 187.7 g a.i. ha^-1^ and 735.7 g a.i. ha^-1^, respectively: **Table 2**). Both values exceeded the recommended 105 g a.i. ha^-1^ field application rate in China.

**Table 2:** Synergistic effects of enzyme inhibitors on cyhalofop-butyl toxicity in different *Leptochloa chinensis* populations.

Population	Treatment	Slope (SE)^a^	ED_50_ (95% CI)^b^(g a.i. ha^-1^)	RR^c^	SR^d^
LC-S	Cyhalofop-butyl	3.55 (0.81)	9.3 (5.1–16.9)	1.0	–
	Cyhalofop-butyl + PBO	1.81 (0.35)	13.6 (8.4–22.1)	–	0.7
	Cyhalofop-butyl + Cyanuric chloride	1.63 (0.12)	11.5 (9.7–13.7)	–	0.8
LC-1701	Cyhalofop-butyl	1.80 (0.30)	187.7 (109.7–321.0)	20.2	–
	Cyhalofop-butyl + PBO	1.04 (0.13)	80.9 (46.0–142.3)	–	2.3
	Cyhalofop-butyl + Cyanuric chloride	1.17 (0.09)	89.7 (64.3–125.2)	–	2.1
LC-1704	Cyhalofop-butyl	1.65 (0.14)	735.7 (628.5–861.3)	79.1	–
	Cyhalofop-butyl + PBO	1.22 (0.12)	497.0 (407.9–605.5)	–	1.5
	Cyhalofop-butyl + Cyanuric chloride	1.22 (0.11)	736.8 (625.5–867.9)	–	1.0

a standard error.

b 95% confidence interval.

c RR (resistance ratio) = cyhalofop-butyl ED_50_ for resistant populations/cyhalofop-butyl ED_50_ for LC-S population.

d SR (synergism ratio) = cyhalofop-butyl ED_50_/ED_50_ of cyhalofop-butyl plus corresponding synergist.

Eight other ACCase-inhibiting herbicides were applied to the LC-1701 and LC-1704 populations to investigate cross-resistance patterns in resistant populations, including aryloxyphenoxypropionates (APP), cyclohexanediones (CHD), and phenylpyrazolines (DEN). LC-1701 and LC-1704 displayed different levels of resistance to APP herbicides such as metamifop, fenoxaprop-*P*-ethyl, haloxyfop-R-methyl, quizalofop-*P*-ethyl, clodinafop-propargyl, and fluzifop-*P*-butyl. The resistance ratios were in the range of 2.1–22.3 (**Table 3**). Compared with susceptible *L. chinensis*, LC-1701 and LC-1704 exhibited enhanced sensitivity to the CHD herbicide sethoxydim and their resistance ratios were 0.5 and 0.1, respectively. Resistance to the DEN herbicide pinoxaden was detected in the LC-1701 population (resistance ratio = 5.8) but not in the LC-1704 population (resistance ratio = 0.6).

**Table 3:** Sensitivity to other ACCase-inhibiting herbicides in *Leptochloa chinensis* populations.

Herbicide	Population	Slope (SE)^a^	ED_50_ (95% CI)^b^(g a.i. ha^-1^)	RR^c^
Metamifop	LC-S	1.21 (0.13)	2.1 (1.4–3.1)	1.0
	LC-1701	1.28 (0.22)	6.0 (3.5–10.4)	2.9
	LC-1704	1.51 (0.35)	12.1 (7.1–20.8)	5.8
Fenoxaprop-*P*-ethyl	LC-S	1.24 (0.30)	0.7 (0.3–1.4)	1.0
	LC-1701	1.40 (0.14)	15.6 (11.9–20.3)	22.3
	LC-1704	1.56 (0.51)	7.7 (2.8–21.3)	11.0
Haloxyfop-R-methyl	LC-S	2.02 (0.33)	1.4 (1.0–2.0)	1.0
	LC-1701	1.58 (0.23)	3.0 (1.9–4.7)	2.1
	LC-1704	1.20 (0.21)	6.2 (3.6–10.8)	4.4
Quizalofop-*P*-ethyl	LC-S	1.90 (0.35)	0.8 (0.5–1.5)	1.0
	LC-1701	2.05 (0.92)	4.4 (1.4–13.6)	5.5
	LC-1704	2.75 (0.36)	3.5 (2.6–4.6)	4.4
Clodinafop-propargyl	LC-S	1.58 (0.24)	0.4 (0.3–0.7)	1.0
	LC-1701	2.19 (0.61)	6.3 (3.1–12.5)	15.8
	LC-1704	1.96 (0.56)	4.6 (2.7–8.0)	11.5
Fluzifop-*P*-butyl	LC-S	1.52 (0.46)	0.7 (0.3–1.4)	1.0
	LC-1701	3.10 (0.46)	8.3 (6.6–10.4)	11.9
	LC-1704	1.01 (0.28)	3.4 (1.5–7.7)	4.9
Sethoxydim	LC-S	0.95 (0.10)	34.4 (22.2–53.3)	1.0
	LC-1701	2.46 (0.50)	16.9 (11.4–24.9)	0.5
	LC-1704	6.91 (2.48)	3.9 (2.3–6.8)	0.1
Pinoxaden	LC-S	0.70 (0.19)	40.4 (16.1–101)	1.0
	LC-1701	1.78 (0.15)	235 (185–298)	5.8
	LC-1704	1.06 (0.07)	24.8 (19.0–32.2)	0.6

a standard error.

b 95% confidence interval.

c RR (resistance ratio) = herbicide ED_50_ for resistant populations/herbicide ED_50_ for LC-S population.

### Sequence analysis and comparison of the ACCase CT domain gene of *L. chinensis*

**Figure 1:**
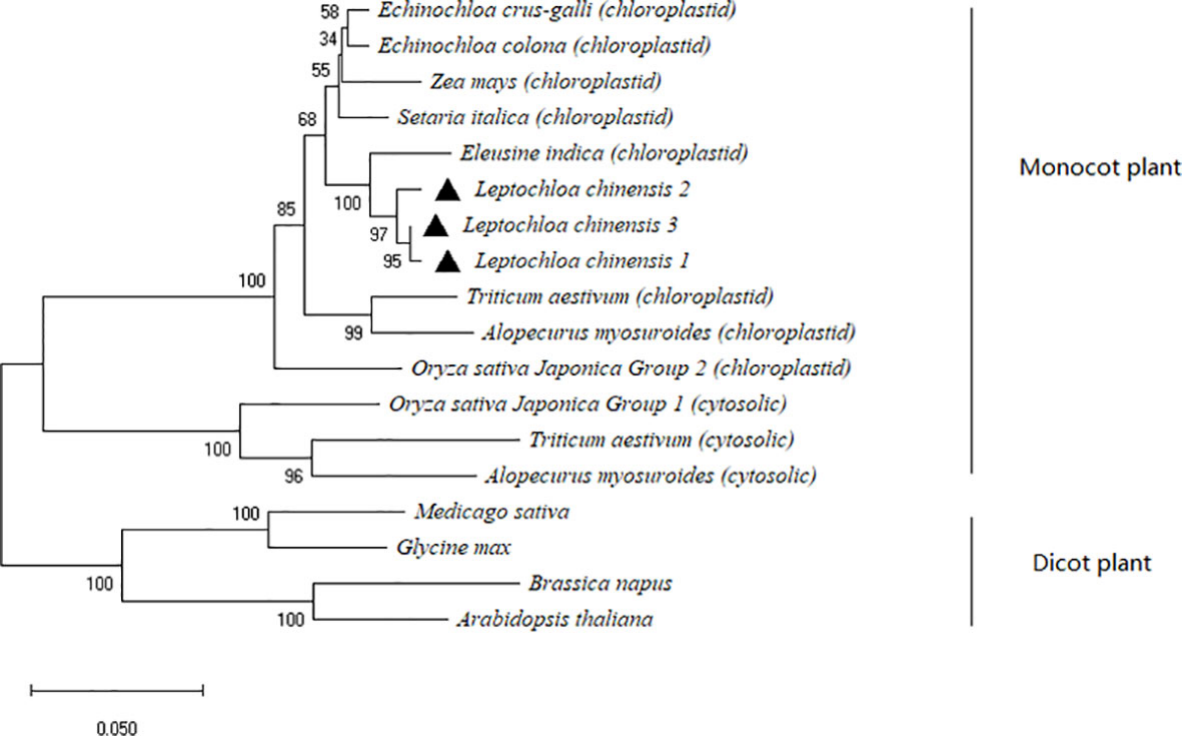
Phylogenetic analysis of the plant ACCase CT domain by the neighbor-joining method. (1) Each of the three types of ACCase CT domain in the susceptible *Leptochloa chinensis* population is labeled by a black triangle. (2) Monocot chloroplastid: *Echinochloa crus-galli* (NCBI accession No. ADR32358); *Echinochloa colona* (NCBI accession No. APZ87886); *Zea mays* (NCBI accession No. AAA80214); *Setaria italica* (NCBI accession No. AAL02056); *Eleusine indica* (NCBI accession No. AHC53984); *Triticum aestivum* (NCBI accession No. AAC39330); *Alopecurus myosuroides* (NCBI accession No. CAC84161); *Oryza sativa Japonica Group 2* (NCBI accession No. B9FK36). (3) Monocot cytosolic: *Oryza sativa Japonica Group 1* (NCBI accession No. Q8S6N5); *Triticum aestivum* (NCBI accession No. AAC49275); *Alopecurus myosuroides* (NCBI accession No. CAF74936). (4) Dicot: *Medicago sativa* (NCBI accession No. AAB42144); *Glycine max* (NCBI accession No. AAA75528); *Brassica napus* (NCBI accession No. CAC19875); *Arabidopsis thaliana* (NCBI accession No. BAA07012).

A 1,929-bp *ACCase* gene fragment was cloned and sequenced for each population. The fragments contained the entire coding region of the CT domain comprising 554 amino acid residues. Alignment of the deduced amino acid sequences revealed three types of ACCase CT domains in the LC-S population which exhibited high homology (> 90%) with the chloroplastic ACCase CT domains of other gramineous weed species (**Figure S1**). Phylogenetic analyses of the plant ACCase CT domains demonstrated that the CT domain gene cloned from the LC-S population clustered with monocot plastidic ACCase (**Figure 1**).

**Figure 2:**
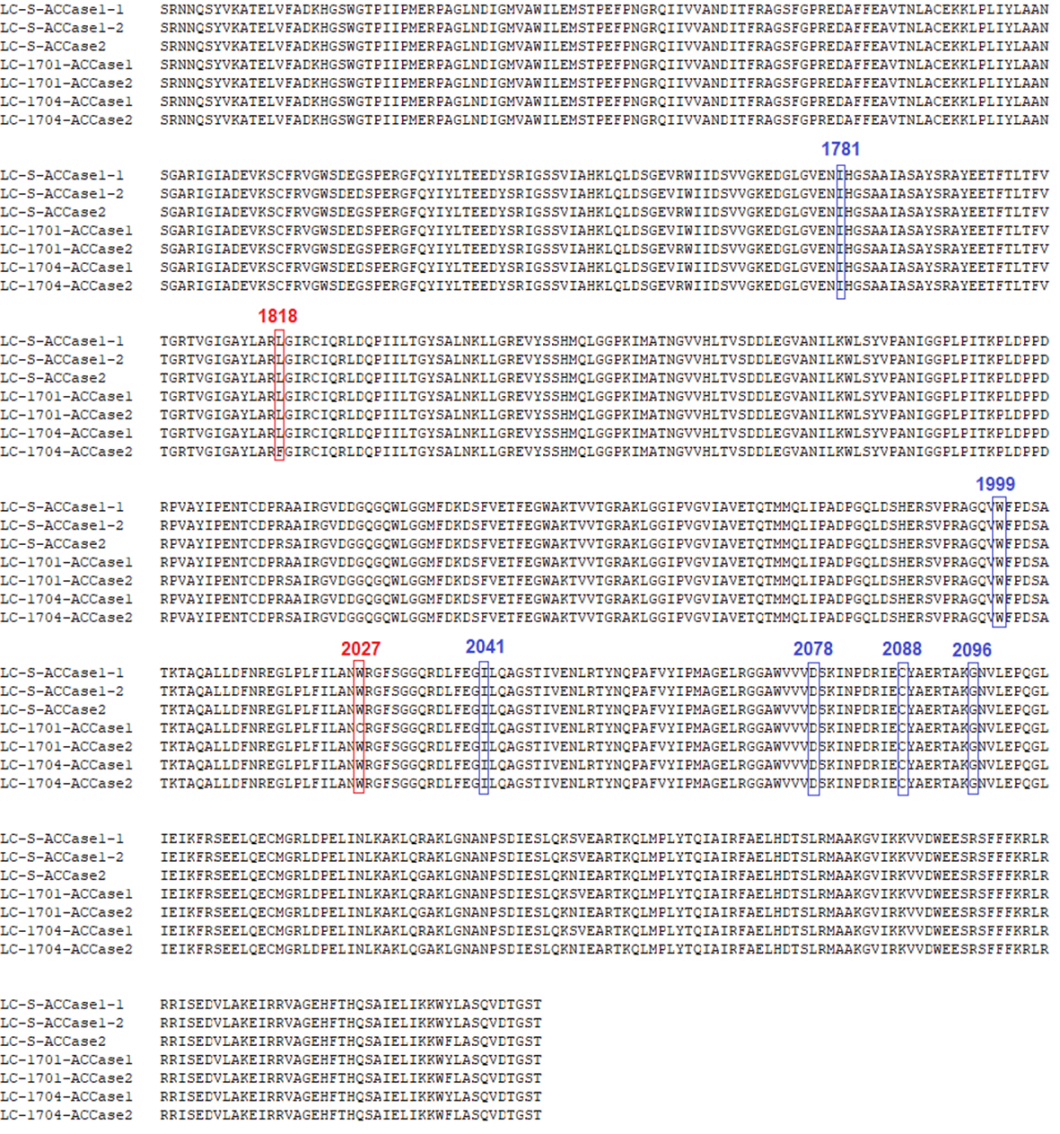
Alignment of the amino acid sequences of partial ACCase in susceptible and resistant *Leptochloa chinensis* populations. Three types of LC-S ACCase are designated as LC-S-ACCase1-1, LC-S-ACCase1-2, and LC-S- ACCase2. Two types of LC-1701 are designated as LC-1701-ACCase1 and LC-1701-*ACCase*2. Two types of LC-1704 are designated as LC-1704-ACCase1 and LC-1704-ACCase2. The red boxed regions represent the mutation sites in the resistant populations. The blue boxed regions represent the other known six resistance-conferring mutation sites.

**Figure 3:**
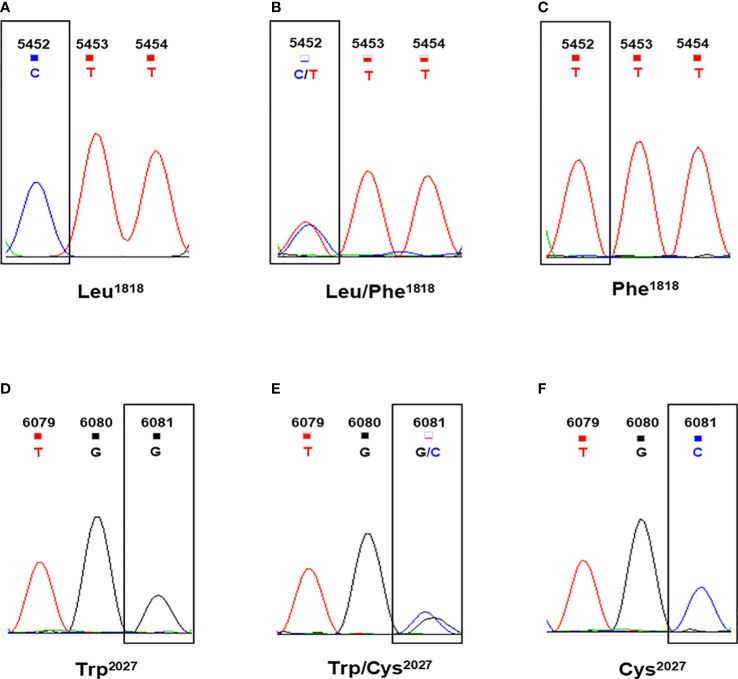
Sequences at codon position 1818 and 2027 of ACCase in *Leptochloa chinensis* individuals. **(A)** The CTT codon for Leu^1818^ in wild-type plants. **(B)** The CTT/TTT codons for Leu/Phe^1818^ in heterozygous mutant plants. **(C)** The TTT codon for Phe^1818^ in homozygous mutant plants. **(D)** The TGG codon for Trp^2027^ in wild-type plants. **(E)** The TGG/TGC codons for Trp/Cys^2027^ in heterozygous mutant plants. **(F)** The TGC codon for Cys^2027^ in homozygous mutant plants.

According to a previous study on *ACCase* sequencing in *L. chinensis* (Deng et al., 2019), further alignment showed that two copies of the *ACCase* gene (*ACCase*1 and *ACCase*2) were successfully cloned from each of the three populations. Regarding the three types of the *ACCase* fragment from LC-S population, they were designated as LC-S-*ACCase*1-1, LC-S-*ACCase*1-2, and LC-S-*ACCase*2. Two types of the *ACCase* fragment were isolated from each resistant population and were designated as LC-1701-*ACCase*1, LC-1701-*ACCase*2, LC-1704-*ACCase*1, and LC-1704-*ACCase*2. Compared with each *ACCase* copy from the LC-S population, the LC-1701-*ACCase*1 presented a Trp^2027^-Cys mutation caused by G6081C, while the LC-1704-*ACCase*2 had a Leu^1818^-Phe mutation caused by C5452T (**Figures 2**, **3**). Additionally, the novel mutation Leu^1818^-Phe was not present in the other four susceptible *L. chinensis* populations reported previously (**Table S5**). The nucleotide and amino acid sites in the ACCase in *L. chinensis* are numbered according to the *A. myosuroides* chloroplastic ACCase.

### Putative cyhalofop-butyl resistance-related mutations in *L. chinensis ACCase*

To analyze the correlations between the detected mutations and cyhalofop-butyl resistance, the point mutations of the target fragment were screened and the polymorphisms in susceptible and resistant *L. chinensis* individuals were identified. In this analysis, the LC-1701 individuals that carried Trp^2027^, Trp/Cys^2027^, and Cys^2027^ were considered as wild-type, heterozygous mutant and homozygous mutant plants, respectively. Similarly, the LC-1704 individuals that carried Leu^1818^, Leu/Phe^1818^, and Phe^1818^ were considered as wild-type, heterozygous mutant, and homozygous mutant plants, respectively (**Figure 3**). As shown in **Table 4**, no mutant alleles at codons 1818 or 2027 were detected in any LC-S individual. For LC-1701, the frequencies of individuals harboring homozygous susceptible (wild type), heterozygous, and homozygous mutant alleles at codon 2027 were 26.7%, 43.3%, and 30.0%, respectively. Similarly, in the LC-1704 population, the mutant allele at codon position 1818 was observed in most individuals, with a frequency of 60.0% and 13.3% for heterozygous and homozygous mutant alleles, respectively.

**Table 4:** Distribution of polymorphism at each ACCase mutation site in different *Leptochloa chinensis* individuals.

Population/Group		Polymorphism frequencies at each amino acid mutation site							
	1818					2027		
	Leu			Leu/Phe	Phe	Trp	Trp/Cys	Cys	
LC-S	100		0		0	100	0	0
LC-1701	ND					26.7	43.3	30.0
LC-1704	26.7		60.0		13.3	ND		
LC-1704H	0		100		0	ND		

ND, no detection.

The Leu^1818^-Phe mutation was never previously associated with cyhalofop-butyl resistance. Hence, the relationship between this novel variant and cyhalofop-butyl resistance in LC-1704 was explored (**Table 4**). With an extremely high resistance level to cyhalofop-butyl, the LC-1704H plants harbored only heterozygous mutant alleles at codon position 1818 and none of them was homozygous susceptible.

**Figure 4:**
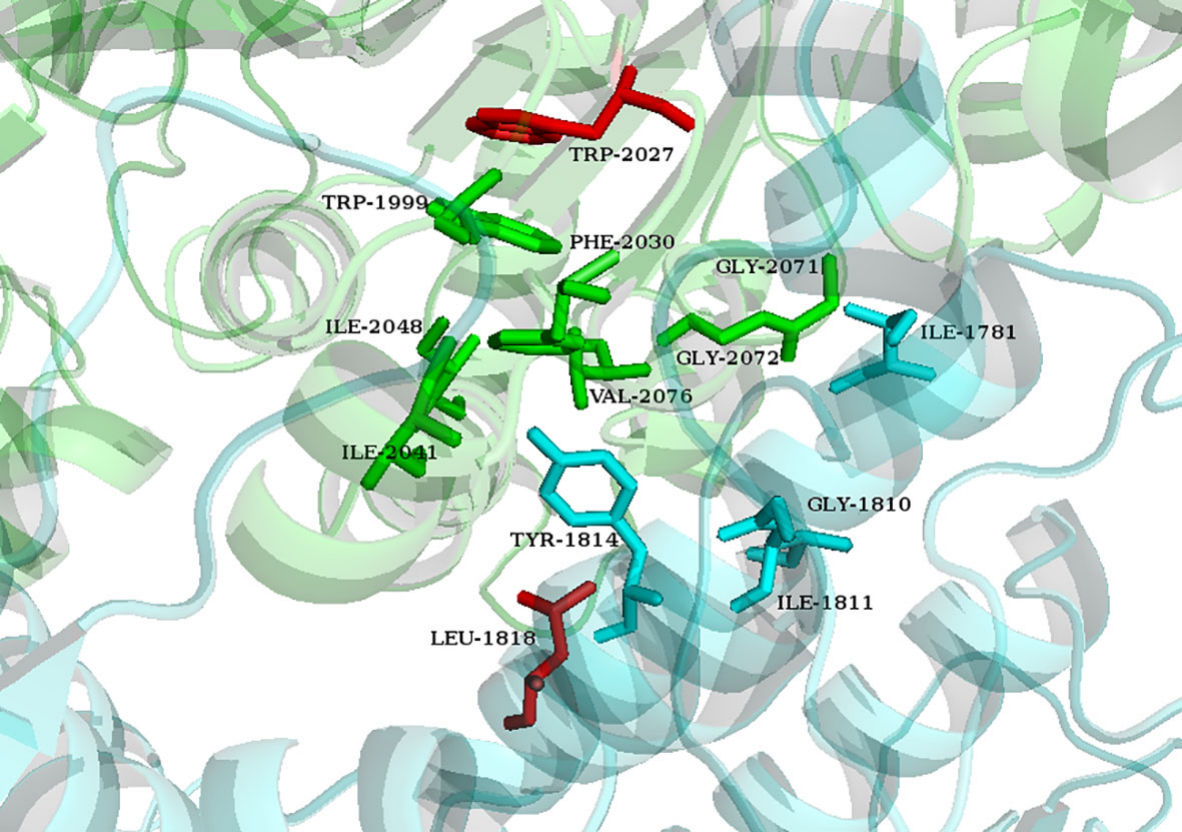
Predicted structure of the substrate-binding pocket in wild-type *Leptochloa chinensis* ACCase. The N and C domains of two ACCase monomers are indicated in blue and green, respectively. Amino acids in the active site region are represented by sticks. Stick color indicates the domain in which the amino acids reside. Two detected mutations are displayed as red sticks.

Based on the crystal structure of the CT domain of yeast ACCase (PDB accession No. 5cte), the 3D structure of the entire CT domain of ACCase of *L. chinensis* was modeled after energy minimization (**Figure 4**). According to the binding mode between yeast ACCase and ACCase-inhibiting herbicides, the active site was located at the interface between the *N* domain of one monomer and the *C* domain of the other monomer in the dimer (Zhang et al., 2004). Thus, it was predicted that the substrate-binding pocket in *L. chinensis* ACCase consisted of Ile^1781^, Gly^1810^, Ile^1811^, Tyr^1814^, Trp^1999`^, Phe^2030`^, Ile^2041`^, Ile^2048`^, Gly^2071`^, Gly^2072`^, and Val^2076`^. The prime (`) symbols indicate the C domains of the other monomers (Zhang et al., 2003). Of note, the two mutation sites detected by our assays (Trp^2027`^ and Leu^1818^) were near the substrate-binding cavity.

### Effects of metabolic inhibitors on cyhalofop-butyl resistance in *L. chinensis*

No synergistic effect of PBO or cyanuric chloride was observed in LC-S (**Table 2**). However, PBO enhanced cyhalofop-butyl toxicity in LC-1701 and LC-1704 and the synergism ratios were 2.3 and 1.5, respectively. Cyanuric chloride significantly suppressed cyhalofop-butyl resistance in LC-1701 but not in LC-1704. In the former population, ED_50_ was reduced to 89.7 g a.i. ha^-1^.

### RNA-Seq quality and DEG analysis

Removal of the low-quality reads left 41,095,172–67,277,694 clean reads from the six RNA libraries for LC-S-C, LC-1701-C, LC-1704-C, LC-S-T, LC-1701-T, and LC-1704-T, with GC content of 53.93%–56.75%. The Q20 and Q30 ranges were 98.40–98.62% and 95.00–95.67%, respectively (**Table S6**). The reference transcriptome was assembled and comprised 71,403 unigenes and 137,173 transcripts (**Table S7**). The mapped ratios of the clean read pairs with the reference transcriptome were in the range of 79.93–83.08% (**Table S8**). Based on the GO, KEGG, COG, NR, Swiss-Prot, and Pfam databases, 71,207 genes were annotated (**Table S9**).

Among the untreated samples, 2,896 and 3,702 DEGs were identified in LC-1701-C *vs*. LC-S-C and LC-1704-C *vs*. LC-S-C, respectively (**Figure S2**). For LC-1701-C *vs*. LC-S-C, there were more downregulated (1,849; 63.8%) than upregulated (1,047; 36.2%) DEGs. For LC-1704-C *vs*. LC-S-C, there were 2,425 (65.5%) downregulated and 1,277 (34.5%) upregulated DEGs. However, the proportions of upregulated and downregulated DEGs were relatively more balanced in the treated samples. There were 6,249 DEGs in LC-1701-T *vs*. LC-S-T, of which 3,083 (49.3%) were upregulated and 3,166 (50.7%) were downregulated. LC-1704-T *vs*. LC-S-T presented with 4,565 (47.5%) upregulated and 5,050 (52.5%) downregulated DEGs.

The functions of the foregoing DEGs were classified by GO annotation (**Figure S3**). Under ‘biological processes’, all DEGs were significantly enriched in metabolic and cellular processes. Under ‘cellular components’, cell and membrane parts were overrepresented in all four groups. Under ‘molecular functions’, the most abundant terms were catalytic activity and binding.

### Validation of putative detoxification enzyme genes mediating cyhalofop-butyl resistance in *L. chinensis*

**Figure 5:**
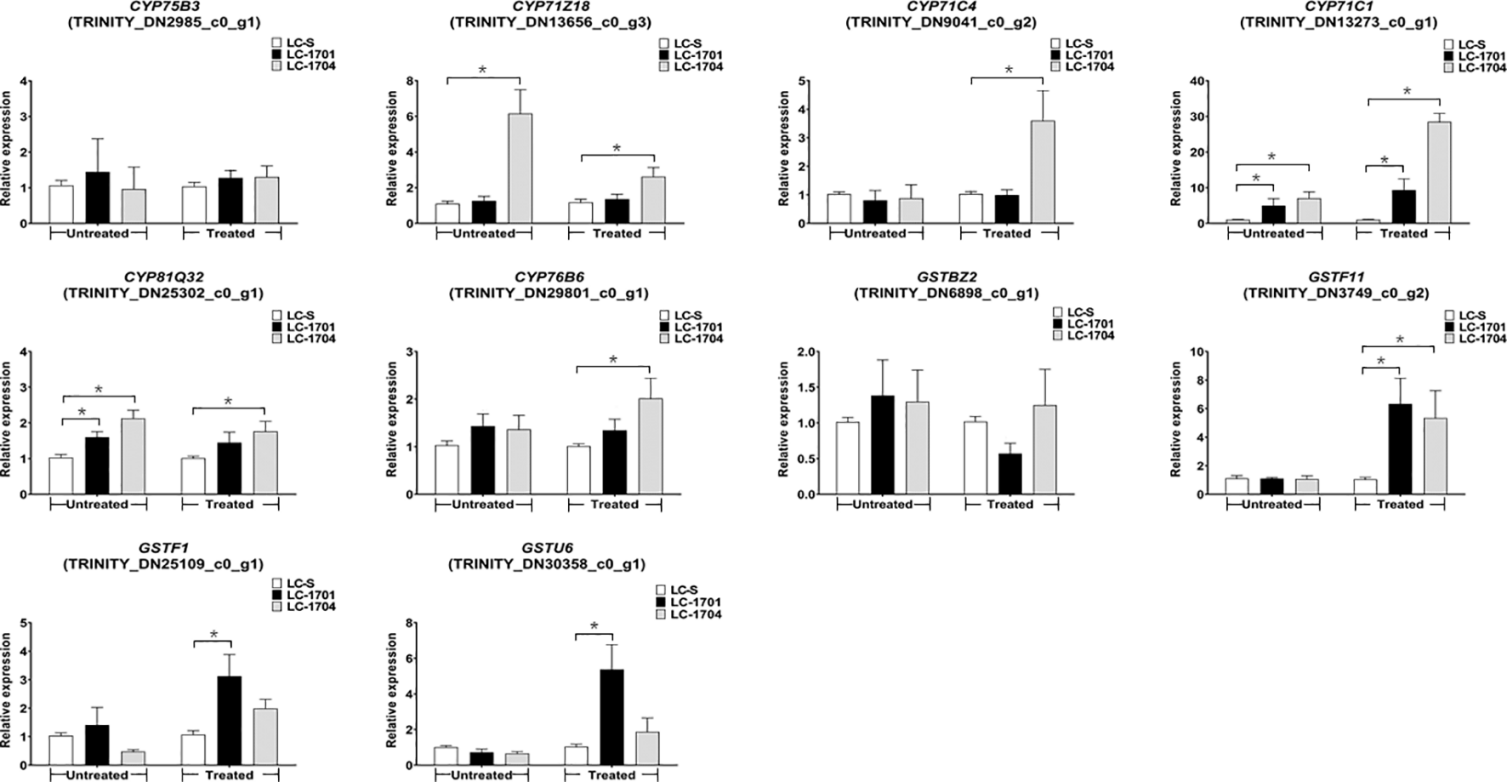
Relative mRNA expression levels of ten candidate genes in susceptible and resistant populations. The relative expression levels shown on the Y-axis were evaluated by the 2^-△△Ct^ method. They represent the ratios of the normalized expression levels of the candidate genes in the resistant population to those of the same genes in the LC-S population. Results for the untreated and cyhalofop-butyl-treated plants grouped on the X-axis are separately analyzed. Error bars indicate the standard error of the mean for three independent replicates. Asterisks above the error bars indicate significant differences between the susceptible and the corresponding resistant populations in each group (one-way ANOVA followed by the LSD test; ^*^ *P* < 0.05).

Based on the foregoing selection criterion, ten differentially expressed contigs were selected to validate the candidate genes responsible for cyhalofop-butyl resistance. These included the six P450s TRINITY_DN2985_c0_g1, TRINITY_DN13656_c0_g3, TRINITY_DN9041_c0_g2, TRINITY_DN13273_c0_g1, TRINITY_DN25302_c0_g1, and TRINITY_DN29801_c0_g1 designated as *CYP75B3, CYP71Z18, CYP71C4, CYP71C1, CYP81Q32*, and *CYP76B6*, respectively (**Table S10**). There were also the four GSTs TRINITY_DN6898_c0_g1, TRINITY_DN3749_c0_g2, TRINITY_DN25109_c0_g1, and TRINITY_DN30358_c0_g1 designated as *GSTBZ2, GSTF11, GSTF1*, and *GSTU6*, respectively (**Table S11**). Using the samples prepared for RNA-Seq and additional samples, mRNA expression patterns of each candidate gene in the susceptible and resistant *L. chinensis* populations were assessed (**Figure 5**). Compared with LC-S, except for *CYP75B3*, the other five P450 genes were significantly upregulated in at least one resistant population. Notably, *CYP71C1* was highly expressed in both resistant populations compared to the susceptible population under normal conditions and after cyhalofop-butyl treatment. Under both conditions, *CYP71Z18* and *CYP81Q32* were upregulated in LC-1704 relative to LC-S, while the latter gene was also constitutively upregulated in LC-1701. However, cyhalofop-butyl-induced *CYP71C4* and *CYP76B6* expression was observed only in LC-1704. Regarding *GST*s, there were no differences in the *GSTBZ2* expression levels between the susceptible and resistant plants. Among the three cyhalofop-butyl-treated populations, *GSTF11*, *GSTF1*, and *GSTU6* were upregulated in LC-1701 whereas *GSTF11* was also upregulated in LC-1704. Thus, five P450 genes (*CYP71Z18*, *CYP71C4*, *CYP71C1*, *CYP81Q32*, and *CYP76B6*) and three GST genes (*GSTF11*, *GSTF1*, and *GSTU6*) were upregulated in the resistant populations.

**Figure 6:**
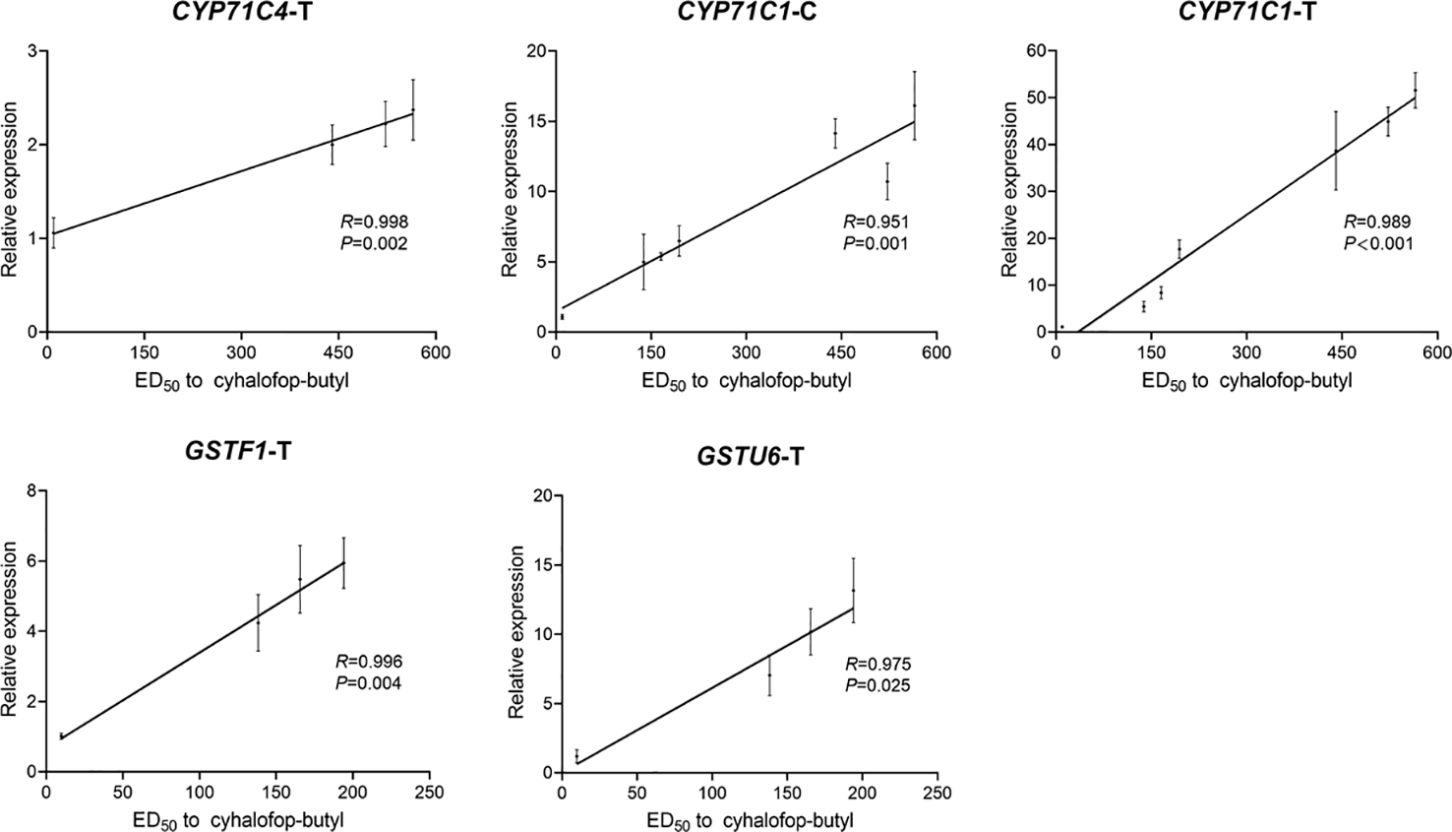
Correlations between the relative expression levels of candidate genes and cyhalofop-butyl resistance levels in F_1_ groups. For the untreated groups, the gene is *CYP71C1*-C. For the cyhalofop-butyl-treated groups, the genes are *CYP71C4*-T, *CYP71C1*-T, *GSTF1*-T, and *GSTU6*-T. Pairwise correlations between gene expression and cyhalofop-butyl ED_50_ were determined by Pearson’s correlation analysis in SPSS v. 20.0. Relative expression levels shown on the Y-axis were evaluated by the 2^-△△Ct^ method. They represent the ratios of the normalized expression levels of the candidate genes in the corresponding group to those of the same genes in the LC-S-F_1_ group. The ED_50_ values for cyhalofop-butyl are shown on the X-axis. Error bars indicate the standard error of the mean for three independent replicates.

Whole-plant dose-response assays and gene expression analyses of the eight candidate genes were conducted on seven F_1_ groups to validate the putative detoxification enzyme genes responsible for cyhalofop-butyl resistance in *L. chinensis*. Compared with the cyhalofop-butyl-susceptible LC-S-F_1_ group (ED_50_ = 9.8 g a.i. ha^-1^), there were various levels of resistance to cyhalofop-butyl in the other six groups (ED_50_ range = 138–565 g a.i. ha^-1^) (**Table S12**). Regarding the expression patterns of the eight candidate genes differed between the two resistant populations, expression of each gene was detected only in the untreated or cyhalofop-butyl-treated samples from certain F_1_ groups (**Tables S13** and **S14**). *CYP71C1* expression was positively correlated with the cyhalofop-butyl resistance levels in both untreated and treated plants (*CYP71C1-*C: *R* = 0.951, *P* = 0.001; *CYP71C1-*T: *R* = 0.989, *P* < 0.001; **Figure 6**). For the LC-1704 F_1_ progeny (LC-1704-F_1_, LC-1704-ED_10_-F_1_, and LC-1704-ED_90_-F_1_), *CYP71C4* expression was strongly correlated with the resistance levels after cyhalofop-butyl treatment (*CYP71C4*-T: *R* = 0.998, *P* = 0.002; **Figure 6**). For the LC-1701 F_1_ progeny (LC-1701-F_1_, LC-1701-ED_10_-F_1_, and LC-1701-ED_90_-F_1_), the *GSTF1* and *GSTU6* expression levels were positively correlated with cyhalofop-butyl resistance (*GSTF1*-T: *R* = 0.996, *P* = 0.004; *GSTU6*-T: *R* = 0.975, *P* = 0.025; **Figure 6**). The expression levels of the other four candidate genes were not significantly correlated with cyhalofop-butyl resistance in *L. chinensis*.

## Discussion

Recently, cross resistance through TSR and NTSR has aggravated ACCase-inhibiting herbicide resistance and lowered the efficacy of weed management strategies (Takano et al., 2021). We investigated cross-resistance patterns in the cyhalofop-butyl-resistant LC-1701 and LC-1704 *L. chinensis* populations. Both had broad cross resistance to APP herbicides but not to the CHD herbicide sethoxydim. These findings were consistent with those of previous studies on cyhalofop-butyl-resistant *L. chinensis* (Yu et al., 2017). However, LC-1701 and LC-1704 differed in terms of their cross-resistance patterns to the DEN herbicide pinoxaden. Hence, the herbicide resistance mechanism of LC-1701 may differ from that of LC-1704 (Beckie and Tardif, 2012). For this reason, we used both LC-1701 and LC-1704 to elucidate the mechanisms of TSR and metabolic herbicide resistance in *L. chinensis*.

TSR is the most common mechanism of resistance to ACCase-inhibiting herbicides (Kaundun, 2014). Consistent with the reported studies (Deng et al., 2019; Deng et al., 2021; Zhao et al., 2022), two copies of the *ACCase* gene were also isolated from each *L. chinensis* population in the current study. The detection of point mutations in the entire ACCase CT domain revealed the occurrences of Trp^2027^-Cys mutation in the ACCase1 of LC-1701 population, and Leu^1818^-Phe mutation in the ACCase2 of LC-1704 population. The subsequent SNP detection in individuals showed these mutations were highly prevalent in the corresponding resistant populations. To the best of our knowledge, herbicide resistance-related ACCase mutations were mainly reported to be located in the ACCase2 of resistant *L. chinensis* (Deng et al., 2019; Zhao et al., 2022), our study is the first report to reveal the occurrence of Trp^2027^-Cys mutation in the *ACCase*1 copy of *L. chinensis*. Since the effects of the Trp^2027^-Cys mutation in *L. chinensis* and other grass weed species are well documented (Liu et al., 2007; Li et al., 2014; Yu et al., 2017; Deng et al., 2019), in this study, further efforts were made to assess the potential associations between cyhalofop-butyl resistance and the novel Leu^1818^-Phe variant in the LC-1704 population. Thus, the LC-1704H group with an extremely high resistance level to cyhalofop-butyl was generated and an additional SNP detection in this group was performed. Considering that individuals with three genotypes at codon position 1818 were detected in the LC-1704 population, if the Leu^1818^-Phe mutation contributes to cyhalofop-butyl resistance, the mutant allele at codon position 1818 should be highly expressed in the LC-1704H group, with no wild-type individuals being detected. Partially consistent with our hypothesis, the SNP detection in LC-1704H showed that all the individuals carried the mutant allele at codon position 1818. However, it was surprising that no individuals bearing homozygous mutants were found in this group. In the SNP detection for both LC-1704 and LC-1704H, the sequencing result showed that all the heterozygotes at position codon 1818 displayed double peaks at the SNP sites which can discriminate two *ACCase* copies in *L. chinensis* (data not shown), revealing that both the *ACCase*1 and *ACCase*2 copies were amplified in these individuals. Since the Leu^1818^-Phe mutation is only detected in the *ACCase*2 of LC-1704, the existence of *ACCase*1 is the most likely reason for the fixed heterozygosity at codon position 1818 in the LC-1704H group. Therefore, the LC-1704H plants can harbor homozygous mutant alleles at codon position 1818 in *ACCase* 2, while the heterozygous genotypes detected in this study are attributed to the so-called homoeologous heterozygotes, which may or may not have the true allelic heterozygosity (Yu et al., 2013). Similar results have been reported in another cyhalofop-butyl-resistant *L. chinensis* population (Zhao et al., 2022), as well as other polyploid weed species (Warwick et al., 2010; Yu et al., 2013). In the current study, sequencing primes used in all SNP detections were designed against conserved regions of the ACCase gene, making it complicated to identify the true allelic heterozygosity at target mutation sites (Yu et al., 2013). As for the resistance mutation sites of *ACCase* in tetraploid *L. chinensis*, it will be of great interest to develop an effective strategy to discriminate true heterozygotes from homoeoallelic heterozygotes. In addition, considering the mutant homozygotes were found in the LC-1704 population but not in the LC-1704H group, it is suggested that NTSR or other unknown mechanisms may play an important role in helping the LC-1704H heterozygotes to survive under the high-dose treatment of cyhalofop-butyl.

Genetic mutations that alter the binding affinity of proteins to herbicides usually occur at or near their catalytic domains or in regions controlling access to these domains (Gaines et al., 2020). Among the seven mutation sites known to confer resistance to ACCase-inhibiting herbicides, Zhang et al. (2004) reported that Trp^1999^, Trp^2027^, and Ile^2041^ residues are clustered near the first aryl ring of haloxyfop. Ile^1781^, Asp^2078^, Cys^2088^, and Gly^2096^ are clustered near each other in the same structure and are distributed around the side chain of the binding pocket (Yu et al., 2010). Computational analyses of these mutations disclosed that Trp^2027^-Cys, Ile^2041^-Asn, Asp^2078^-Gly, and Gly^2096^-Ala may cause substantial conformational changes in the binding pocket. Thus, plants with the foregoing mutations may have decreased herbicide sensitivity (Zhu et al., 2009). Our homology models showed that Leu^1818^ and Trp^2027^ were localized near the binding pocket in *L. chinensis.* Hence, they might influence binding between herbicides and ACCase. However, the effects of these detected mutations in conferring cyhalofop-butyl resistance, especially regarding the novel Leu^1818^-Phe, remain unclear and warrant further research.

Weeds with metabolism-based resistance can detoxify herbicides and are, therefore, difficult to control (Yu and Powles, 2014). P450s and GSTs are involved mainly in the first and second phases of xenobiotic detoxification, respectively. Upregulation of P450s and GSTs confers ACCase-inhibiting herbicide resistance in various weed species (Kaundun, 2014). In cases focused on herbicide resistance mechanisms, detoxification enzyme inhibitors are usually used to explore P450- and GST-mediated metabolic resistance in weeds. Wang et al. (2013) reported that a P450 inhibitor reversed fenoxaprop-*P*-ethyl resistance in *Poa annua*, suggesting P450-mediated herbicide resistance in this weed species. Cummins et al. (2013) reported the GST inhibitor synergized with ACCase-inhibiting herbicide in multiple-resistant *A. myosuroides* populations, indicating the role of enhanced GSTs as a resistance mechanism. The results of our study corroborated those of a previous report on cyhalofop-butyl-resistant *L. chinensis* (Zhao et al., 2022). We found that PBO application increased cyhalofop-butyl toxicity in two resistant populations but had no effect on the susceptible population. This discovery furnishes additional evidence that P450s are implicated in cyhalofop-butyl resistance in *L. chinensis*. However, the synergistic effect of cyanuric chloride was only detected in the LC-1701 population. For this reason, GSTs may only mediate cyhalofop-butyl resistance in certain specific populations. Our synergy assays showed that P450s and GSTs potentially metabolize cyhalofop-butyl in resistant *L. chinensis* and revealed different metabolic resistance mechanisms between two cyhalofop-butyl-resistant populations. The foregoing findings provided a basis for subsequent screening of candidate genes regulating metabolic herbicide resistance.

Various omics platforms have been used to clarify the molecular mechanisms of herbicide resistance (Maroli et al., 2018). Transcriptomics have been widely utilized to elucidate NTSR mechanisms in several gramineous weed species such as *A. myosuroides* (Gardin et al., 2015), *Lolium rigidum* (Gaines et al., 2014), and *Beckmannia syzigachne* (Wang et al., 2022a). In the current study, we subjected two resistant *L. chinensis* populations from Zhejiang Province to transcriptome analysis and expression validation. We identified five *P450*s and three *GST*s as candidates for metabolic resistance in *L. chinensis* as each gene was highly expressed in at least one resistant population. Further exploration showed that the constitutive and inducible *CYP71C1* expression patterns were positively correlated with the cyhalofop-butyl resistance levels. Consequently, *CYP71C1* was the most likely candidate responsible for cyhalofop-butyl resistance in *L. chinensis*. *CYP71C4*, *GSTF1*, and *GSTU6* were also promising cyhalofop-butyl-tolerance genes based on their resistance-associated expression patterns in response to herbicide treatment. Considering three resistance-related P450 genes, namely, one *CYP76* and two *CYP71A*s, were identified in a cyhalofop-butyl-resistant population in Hunan Province (Chen et al., 2021), our findings expand the candidate gene pool implicated in metabolic herbicide resistance and lay the foundation for future investigations clarifying cyhalofop-butyl NTSR in *L. chinensis*.

It was recently confirmed that certain plant P450 enzymes in the CYP71 family metabolize herbicides. Hence, they might participate in herbicide resistance (Gaines et al., 2020). Siminszky et al. (1999) demonstrated the capacity of CYP71A10 to metabolize phenylurea herbicides in *Glycine max*, and its contribution in linuron resistance was subsequently confirmed (Siminszky et al., 2000). The activity of the herbicide-metabolizing CYP71 was also demonstrated in *Triticum aestivum* (Xiang et al., 2006), *Arabidopsis thaliana* (Hayashi et al., 2007), and *Nicotiana tabacum* (Yamada et al., 2000). However, CYP71s have high substrate specificity (Dimaano and Iwakami, 2021) and their capacity to metabolize herbicides has not yet been confirmed for grass weed species. With respect to plant GSTs, phi-class GSTFs and tau-class GSTUs have been most commonly reported to detoxify herbicides in crop plants (Cummins et al., 2011). Interestingly, instead of decreasing sensitivity to herbicides by detoxification, *Am*GSTF1 acts as a glutathione peroxidase to accumulate protective flavonoids in resistant *A. myosuroides* (Cummins et al., 2013). The foregoing discoveries suggested that various metabolic pathways may be involved in enhancing GST-mediated herbicide tolerance in plants. Regarding the four candidate genes identified to responsible for metabolic resistance, future research should endeavor to determine their exact roles in resistance to cyhalofop-butyl and other ACCase-inhibiting herbicides in *L. chinensis*, such as examining their herbicide-metabolising activities or other biological functions related to herbicide resistance. Considering the complexity of this resistance mechanism, besides P450s and GSTs, genes from other enzyme families involved in metabolic resistance to cyhalofop-butyl could not be considered in this study (Gaines et al., 2020). Therefore, further investigation of these metabolic pathways is required to identify the metabolites involved in this process. Comparing those metabolites’ abundance among different *L. chinensis* populations across time points may provide a more detailed description of metabolism-based mechanisms in cyhalofop-butyl-resistant *L. chinensis* and their relationship with plant stress response pathways.

## Conclusion

Some of the molecular mechanisms involving TSR and metabolism-based resistance were successfully described using two cyhalofop-butyl-resistant *L. chinensis* populations. Regarding the LC-1701 population, the known mutation Trp^2027^-Cys in the chloroplastic ACCase CT domain was detected, and three metabolism-related genes (*CYP71C1*, *GSTF1*, and *GSTU6*) may confer cyhalofop-butyl resistance. In contrast, rather than this reported mutation, a so-far unknown non-synonymous substitution (Leu^1818^-Phe) was first discovered in the LC-1704 population. Moreover, the P450 genes *CYP71C1* and *CYP71C4* were identified as candidate genes responsible for cyhalofop-butyl resistance in this population. Differences in resistance mechanisms between the two resistant populations highlighted the diversity of involved herbicide resistance mechanisms within a single species, possibly leading to different cross-resistance patterns to ACCase-inhibiting herbicides. Ultimately, these findings provide a sound theoretical basis upon which further research into the underlying mechanisms of cyhalofop-butyl resistance can be conducted and aid in the development of scientific approaches to integrated management of herbicide-resistant populations.

## Data availability statement

The original contributions presented in the study are publicly available. This data can be found here: NCBI, PRJNA857146 and GenBank OP056462-OP056466.

## Author contributions

CW contributed to the conception and design of this research. YZ, LC, WS, TC, and MX conducted experiments and analyzed the data. YZ wrote the main manuscript text. All authors contributed to the article and approved the submitted version.

## Funding

This work was supported by the Natural Science Foundation of Zhejiang Province (Grant No. LQ20C140005), and the National Key Research and Development Plan of China (Grant No. 2016YFD0200800).

## Acknowledgments

We thank the Natural Science Foundation of Zhejiang Province and the National Key Research and Development Plan of China for the financial support of this study. We also thank Editage for the English language editing.

## Conflict of interest

The authors declare that the research was conducted in the absence of any commercial or financial relationships that could be construed as a potential conflict of interest.

## Publisher’s note

All claims expressed in this article are solely those of the authors and do not necessarily represent those of their affiliated organizations, or those of the publisher, the editors and the reviewers. Any product that may be evaluated in this article, or claim that may be made by its manufacturer, is not guaranteed or endorsed by the publisher.

## Abbreviations

ACCase–acetyl-CoA carboxylase; APP–aryloxyphenoxypropionate; CHD–cyclohexanedione; COG–Clusters of Orthologous Groups; CT–carboxyltransferase; DAT–day after treatment; DEG–differentially expressed gene; DEN–phenylpyrazoline; EC–emulsifiable concentrate; ED_10_–10% effective dose; ED_50_–50% effective dose; ED_90_–90% effective dose; EW–emulsion in water; GO–Gene Ontology; GST–glutathione *S*-transferase; KEGG–Kyoto Encyclopedia of Genes and Genomes; ME–microemulsion; NR–non-redundant protein database; NTSR–non-target-site resistance; TSR–target-site resistance; PBO–piperonyl butoxide; P450–cytochrome P450 monooxygenase; qRT-PCR–quantitative reverse-transcription polymerase chain reaction; RNA-Seq–RNA sequencing; SNP–single nucleotide polymorphism.
