# Supplementary material for: Diverse mechanisms associated with cyhalofop-butyl resistance in Chinese sprangletop (Leptochloa chinensis (L.) Nees): Characterization of target-site mutations and metabolic resistance-related genes in two resistant populations
Source: Front Plant Sci. 2022 Nov 28;13:990085. doi: 10.3389/fpls.2022.990085 (PMC9742530; doi:10.3389/fpls.2022.990085)
Supplement: Supplementary file 1 [file DataSheet_1.docx]

Supplementary Material

| **Table S1.** Doses of ACCase-inhibiting herbicides applied for the bioassay. | | | |
| --- | --- | --- | --- |
| Herbicide | Dose (g a.i. ha^-1^) | | |
|  | LC-S | LC-1701 | LC-1704 |
| Cyhalofop-butyl | 3, 6, 12, 24, 48, 96 | 78, 118, 179, 294, 458, 732 | 179, 294, 458, 732, 1172, 1875 |
| Metamifop | 0.35, 0.70, 1.41, 2.82, 5.62, 11.2 | 1.76, 3.51, 7.04, 14.1, 28.2, 56.2 | 1.76, 3.51, 7.03, 14.1 28.2, 56.2 |
| Fenoxaprop-*P*-ethyl | 0.097, 0.20, 0.39, 0.77, 1.55, 3.10 | 0.97, 1.95, 3.88, 7.73, 15.5, 31.0 | 0.49, 0.97, 1.95, 3.88, 7.73, 15.5 |
| Haloxyfop-R-methyl | 0.15, 0.31, 0.61, 1.21, 2.43, 4.86 | 0.76, 1.52, 3.05, 6.07, 12.1, 24.3 | 0.31, 0.61, 1.21, 2.43, 4.86, 9.72 |
| Quizalofop-*P*-ethyl | 0.071, 0.14, 0.28, 0.56, 1.12 | 0.44, 0.88, 1.76, 3.51, 7.04 | 0.35, 0.71, 1.41, 2.82, 5.62 |
| Clodinafop-propargyl | 0.21, 0.42, 0.84, 1.68, 3.38 | 1.06, 2.12, 4.23, 8.43, 16.8 | 0.84, 1.68, 3.38, 6.75, 13.5 |
| Fluzifop-*P*-butyl | 0.11, 0.21, 0.42, 0.84, 1.68, 3.38 | 1.06, 2.12, 4.23, 8.43, 16.8, 33.8 | 0.53, 1.06, 2.12, 4.23, 8.43, 16.8 |
| Sethoxydim | 3.52, 7.03, 14.1, 28.1, 56.2 | 3.52, 7.03, 14.1, 28.1, 56.2 | 1.76, 3.52, 7.03, 10.1, 14.1 |
| Pinoxaden | 12.0, 54.0, 60.0, 270, 300 | 18.0, 54.0, 90.0, 270, 450 | 2.40, 12.0, 60.0, 270, 300 |

| **Table S2.** Primer information for candidate genes used in qRT-RCR. | | | |
| --- | --- | --- | --- |
| Contig  (Swiss-Prot description) | Primer (5'-3') | Length (bp) | PCR efficiency  (%) |
| TRINITY_DN2985_c0_g1  (CYP75B3) | Forward: GACCTCCCGCGCCTCAC | 220 | 101.8 |
|  | Reverse: GGAGGAAGCGGTCGGGG |  |  |
| TRINITY_DN13656_c0_g3  (CYP71Z18) | Forward: GATCGAGCAGCACCAGGA | 231 | 97.5 |
|  | Reverse: CTCCTCATCACGTCAGGGTTC |  |  |
| TRINITY_DN9041_c0_g2  (CYP71C4) | Forward: TGGTCCTCGAGGTTGCC | 157 | 101.1 |
|  | Reverse: GTCTCCTTGATCACTGCCTTGAG |  |  |
| TRINITY_DN13273_c0_g1  (CYP71C1) | Forward: CACTAATGACAACGTGTGCCG | 206 | 97.4 |
|  | Reverse: CGTCCCACCTCTTGCGAAG |  |  |
| TRINITY_DN25302_c0_g1  (CYP81Q32) | Forward: TCGGCACAACACGTCTAGTAGA | 204 | 104.4 |
|  | Reverse: TGGGGTCACGATGGATTGC |  |  |
| TRINITY_DN29801_c0_g1  (CYP76B6) | Forward: AGGTCGCAGGAGTTCAAACA | 83 | 97.7 |
|  | Reverse: ATCGCCGGGAAGAAGTCTG |  |  |
| TRINITY_DN6898_c0_g1  (GSTBZ2) | Forward: CTTCCTCCTCGCAGTCTCCTAGT | 116 | 100.7 |
|  | Reverse: CCAGAACCTGTCGTCGATGA |  |  |
| TRINITY_DN3749_c0_g2  (GSTF11) | Forward: GCCACATCACCTTCGTCGAG | 199 | 99.9 |
|  | Reverse: GTGGGCAGGTAAGAGAGGCTG |  |  |
| TRINITY_DN25109_c0_g1  (GSTF1) | Forward: GAGAGAAGGAGAAGGCAACCC | 217 | 98.3 |
|  | Reverse: GCTCTTGGACAGGCGTTCC |  |  |
| TRINITY_DN30358_c0_g1  (GSTU6) | Forward: TACCTCGACGATGCCTTCCC | 222 | 100.3 |
|  | Reverse: CCCGGAGAGCTCGACGAAC |  |  |

| **Table S3.** Expression patterns of candidate genes in two resistant population. | | | | |
| --- | --- | --- | --- | --- |
| Candidate genes | LC-1071^a^ | | LC-1074^a^ | |
|  | C^b^ | T^c^ | C^b^ | T^c^ |
| *CYP71Z18* |  |  | √ | √ |
| *CYP71C4* |  |  |  | √ |
| *CYP71C1* | √ | √ | √ | √ |
| *CYP81Q32* | √ |  | √ | √ |
| *CYP76B6* |  |  |  | √ |
| *GSTF11* |  | √ |  | √ |
| *GSTF1* |  | √ |  |  |
| *GSTU6* |  | √ |  |  |
| ^a^√: up-regulated gene in the resistant population relative to the susceptible population under the corresponding condition.  ^b^C: without cyhalofop-butyl treatment.  ^c^T: with cyhalofop-butyl treatment. | | | | |

| **Table S4.** F_1_ progeny used for further qRT-PCR of each candidate gene. | | | | | | | | | | | | | | |
| --- | --- | --- | --- | --- | --- | --- | --- | --- | --- | --- | --- | --- | --- | --- |
| Candidate genes | LC-S-F_1_^a^ | | LC-1701-F_1_^a^ | | LC-1701-ED_10_-F_1_^a^ | | LC-1701-ED_90_-F_1_^a^ | | LC-1704-F_1_^a^ | | LC-1704-ED_10_-F_1_^a^ | | LC-1704-ED_90_-F_1_^a^ | |
|  | C^b^ | T^c^ | C^b^ | T^c^ | C^b^ | T^c^ | C^b^ | T^c^ | C^b^ | T^c^ | C^b^ | T^c^ | C^b^ | T^c^ |
| *CYP71Z18* | √ | √ |  |  |  |  |  |  | √ | √ | √ | √ | √ | √ |
| *CYP71C4* |  | √ |  |  |  |  |  |  |  | √ |  | √ |  | √ |
| *CYP71C1* | √ | √ | √ | √ | √ | √ | √ | √ | √ | √ | √ | √ | √ | √ |
| *CYP81Q32* | √ | √ | √ |  | √ |  | √ |  | √ | √ | √ | √ | √ | √ |
| *CYP76B6* |  | √ |  |  |  |  |  |  |  | √ |  | √ |  | √ |
| *GSTF11* |  | √ |  | √ |  | √ |  | √ |  |  |  |  |  |  |
| *GSTF1* |  | √ |  | √ |  | √ |  | √ |  |  |  |  |  |  |
| *GSTU6* |  | √ |  | √ |  | √ |  | √ |  |  |  |  |  |  |
| ^a^√: selected F_1_ progeny for further qRT-PCR of each candidate gene under the corresponding condition.  ^b^C: without cyhalofop-butyl treatment.  ^c^T: with cyhalofop-butyl treatment. | | | | | | | | | | | | | | |

| **Table S5**. Amino acid at the position 1818 of different ACCase in susceptible and resistant *Leptochloa chinensis* populations. | | | |
| --- | --- | --- | --- |
| Biotype | Population^b^ | Amino acid at the position 1818 ^a^ | |
|  |  | ACCase 1 | ACCase 2 |
| Susceptible | JS1 | Leu | Leu |
|  | YZ-S | Leu | Leu |
|  | SJ3-1 | Leu | NR |
|  | S3 | Leu | NR |
|  | LC-S | Leu | Leu |
| Resistant | LC-1701 | Leu | Leu |
|  | LC-1704 | Leu | Phe |
| ^a^NR: no reported.  ^b^Sequence information source of each susceptible population: JS1 and YZ-S (NCBI accession No. MW266986; Deng et al., 2019; Deng et al., 2021); SJ3-1 (NCBI accession No. MT394618; Yuan et al., 2021); S3 (NCBI accession No. AY662693). | | | |

| **Table S6.** Data of clean reads from different *Leptochloa chinensis* samples. | | | | | | |
| --- | --- | --- | --- | --- | --- | --- |
| Sample | Clean reads | Clean bases | Error rate  (%) | Q20  (%) | Q30  (%) | GC content  (%) |
| LC-S-C_1 | 60439948 | 8980193055 | 0.0238 | 98.52 | 95.35 | 55.80 |
| LC-S-C_2 | 67277694 | 9980070393 | 0.024 | 98.44 | 95.14 | 56.04 |
| LC-S-C_3 | 59986928 | 8916889773 | 0.0237 | 98.55 | 95.43 | 56.71 |
| LC-1701-C_1 | 59740634 | 8867636965 | 0.0236 | 98.59 | 95.56 | 56.05 |
| LC-1701-C_2 | 57924956 | 8611236389 | 0.0239 | 98.48 | 95.20 | 55.93 |
| LC-1701-C_3 | 52411372 | 7777363915 | 0.0238 | 98.54 | 95.39 | 55.10 |
| LC-1704-C_1 | 49548524 | 7370306522 | 0.0237 | 98.55 | 95.40 | 56.22 |
| LC-1704-C_2 | 61608330 | 9160205956 | 0.0238 | 98.53 | 95.34 | 56.75 |
| LC-1704-C_3 | 55337780 | 8257111686 | 0.0241 | 98.40 | 95.00 | 56.52 |
| LC-S-T_1 | 55634864 | 8265460828 | 0.024 | 98.46 | 95.12 | 54.55 |
| LC-S-T_2 | 49933186 | 7422656679 | 0.0238 | 98.55 | 95.35 | 54.28 |
| LC-S-T_3 | 58318846 | 8663863634 | 0.0239 | 98.49 | 95.23 | 55.20 |
| LC-1701-T_1 | 47394904 | 6990133436 | 0.024 | 98.43 | 95.10 | 54.84 |
| LC-1701-T_2 | 50141712 | 7479446808 | 0.0239 | 98.49 | 95.23 | 54.66 |
| LC-1701-T_3 | 47244424 | 7031979439 | 0.024 | 98.46 | 95.17 | 54.59 |
| LC-1704-T_1 | 41095172 | 6108797701 | 0.0238 | 98.51 | 95.39 | 55.72 |
| LC-1704-T_2 | 50135874 | 7290059896 | 0.0236 | 98.57 | 95.53 | 53.93 |
| LC-1704-T_3 | 43168224 | 6333432855 | 0.0235 | 98.62 | 95.67 | 55.23 |

| **Table S7.** Information of the assembled reference transcriptome for RNA sequencing in *Leptochloa chinensis*. | | |
| --- | --- | --- |
| Type | Unigene | Transcript |
| Total number | 71403 | 137173 |
| Total base | 75376719 | 185493252 |
| Largest length (bp) | 15672 | 15672 |
| Smallest length (bp) | 201 | 201 |
| Average length (bp) | 1055.65 | 1352.26 |
| N50 length (bp) | 1833 | 2052 |
| E90N50 length (bp) | 2820 | 2207 |
| Fragment mapped percent(%) | 55.51 | 77.931 |
| GC percent (%) | 47.55 | 48.26 |
| TransRate score | 0.25853 | 0.37387 |
| BUSCO score | C:77.2% [S:72.8%; D:4.4%] | C:92.5% [S:20.1%; D:72.4%] |

| **Table S8.** Mapping results of clean reads from each *Leptochloa chinensis* sample to the reference transcriptome. | | | |
| --- | --- | --- | --- |
| Sample | Clean reads  (Pair reads) | Mapped reads | Mapped ratio |
| LC-S-C_1 | 30219974 | 24384304 | 80.69% |
| LC-S-C_2 | 33638847 | 27652062 | 82.20% |
| LC-S-C_3 | 29993464 | 24470650 | 81.59% |
| LC-1701-C_1 | 29870317 | 24483966 | 81.97% |
| LC-1701-C_2 | 28962478 | 23592493 | 81.46% |
| LC-1701-C_3 | 26205686 | 21629844 | 82.54% |
| LC-1704-C_1 | 24774262 | 20442002 | 82.51% |
| LC-1704-C_2 | 30804165 | 25295717 | 82.12% |
| LC-1704-C_3 | 27668890 | 22497402 | 81.31% |
| LC-S-T_1 | 27817432 | 22740170 | 81.75% |
| LC-S-T_2 | 24966593 | 20503801 | 82.12% |
| LC-S-T_3 | 29159423 | 23925970 | 82.05% |
| LC-1701-T_1 | 23697452 | 19632534 | 82.85% |
| LC-1701-T_2 | 25070856 | 20349566 | 81.17% |
| LC-1701-T_3 | 23622212 | 19564523 | 82.82% |
| LC-1704-T_1 | 20547586 | 16423145 | 79.93% |
| LC-1704-T_2 | 25067937 | 20669769 | 82.46% |
| LC-1704-T_3 | 21584112 | 17933024 | 83.08% |

| **Table S9.**  Functional annotation of unigenes in *Leptochloa chinensis* transcriptome. | | |
| --- | --- | --- |
| Database | Unigene number | Percentage (%) |
| GO | 22908 | 32.2 |
| KEGG | 17891 | 25.1 |
| COG | 38702 | 54.4 |
| NR | 24756 | 34.8 |
| Swiss-Prot | 28587 | 40.2 |
| Pfam | 27273 | 38.3 |
| Total | 71207 | 100 |

| **Table S10.** RNA-Seq information of candidate DEGs for P450s. | | | | | | | |
| --- | --- | --- | --- | --- | --- | --- | --- |
| Contig  (Swiss-Prot description) | Compared group^a^ | FC | Log_2_FC | *P* value | *P* adjust | Significant^b^ | Regulate |
| TRINITY_DN2985_c0_g1  (CYP75B3) | 1 | 5.97 | 2.58 | 5.78×10^-11^ | 1.27×10^-8^ | yes | up |
|  | 2 | 6.94 | 2.80 | 1.03×10^-7^ | 4.29×10^-6^ | yes | up |
|  | 3 | 3.75 | 1.91 | 0.00016 | 0.0011 | yes | up |
|  | 4 | 6.27 | 2.65 | 1.96×10^-9^ | 3.26×10^-8^ | yes | up |
| TRINITY_DN13656_c0_g3  (CYP71Z18) | 1 | 4.42 | 2.14 | 0.00016 | 0.0038 | yes | up |
|  | 2 | 9.44 | 3.24 | 9.89×10^-9^ | 5.34×10^-7^ | yes | up |
|  | 3 | 3.85 | 1.94 | 1.25×10^-13^ | 5.30×10^-12^ | yes | up |
|  | 4 | 7.74 | 2.95 | 9.28×10^-11^ | 1.91×10^-9^ | yes | up |
| TRINITY_DN9041_c0_g2  (CYP71C4) | 1 | 3.93 | 1.97 | 0.013 | 0.10 | no | up |
|  | 2 | 12.90 | 3.69 | 7.39×10^-6^ | 0.00018 | yes | up |
|  | 3 | 2.74 | 1.46 | 0.19 | 0.36 | no | up |
|  | 4 | 12.72 | 3.67 | 3.38×10^-5^ | 0.00023 | yes | up |
| TRINITY_DN13273_c0_g1  (CYP71C1) | 1 | 0.07 | -3.76 | 1.13×10^-6^ | 6.41×10^-5^ | yes | down |
|  | 2 | 3.24 | 1.70 | 2.17×10^-7^ | 8.42×10^-6^ | yes | up |
|  | 3 | 0.95 | -0.08 | 0.89 | 0.94 | no | down |
|  | 4 | 12.05 | 3.59 | 3.32×10^-11^ | 7.31×10^-10^ | yes | up |
| TRINITY_DN25302_c0_g1  (CYP81Q32) | 1 | 1.52 | 0.61 | 0.098 | 0.35 | no | up |
|  | 2 | 1.13 | 0.18 | 0.59 | 0.81 | no | up |
|  | 3 | 2.30 | 1.20 | 0.0014 | 0.0068 | yes | up |
|  | 4 | 2.32 | 1.21 | 0.0060 | 0.022 | yes | up |
| TRINITY_DN29801_c0_g1  (CYP76B6) | 1 | 1.60 | 0.68 | 0.070 | 0.29 | no | up |
|  | 2 | 1.96 | 0.97 | 0.0064 | 0.042 | no | up |
|  | 3 | 2.93 | 1.55 | 9.71×10^-10^ | 2.02×10^-8^ | yes | up |
|  | 4 | 2.71 | 1.44 | 0.00013 | 0.00080 | yes | up |
| ^a^Compared group 1: LC-1701-C vs. LC-S-C; Compared group 2: LC-1704-C vs. LC-S-C; Compared group 3: LC-1701-T vs. LC-S-T; Compared group 4: LC-1704-T vs. LC-S-T.  ^b^Genes with an absolute value of log_2_fold-change (FC)≥1 and a *p*-value＜0.05 were considered differently expressed. | | | | | | | |

| **Table S11.** RNA-Seq information of candidate DEGs for GSTs. | | | | | | | |
| --- | --- | --- | --- | --- | --- | --- | --- |
| Contig  (Swiss-Prot description) | Compared group^a^ | FC | Log_2_FC | *P* value | *P* adjust | Significant^b^ | Regulate |
| TRINITY_DN6898_c0_g1  (GSTBZ2) | 1 | 5.62 | 2.49 | 3.68×10^-6^ | 0.00017 | yes | up |
|  | 2 | 2.11 | 1.07 | 0.0097 | 0.057 | no | up |
|  | 3 | 0.82 | -0.28 | 0.46 | 0.64 | no | down |
|  | 4 | 0.96 | -0.06 | 0.88 | 0.92 | no | down |
| TRINITY_DN3749_c0_g2  (GSTF11) | 1 | 1.27 | 0.34 | 0.34 | 0.68 | no | up |
|  | 2 | 0.71 | -0.49 | 0.18 | 0.43 | no | down |
|  | 3 | 2.57 | 1.36 | 0.0014 | 0.0071 | yes | up |
|  | 4 | 2.14 | 1.10 | 0.032 | 0.083 | no | up |
| TRINITY_DN25109_c0_g1  (GSTF1) | 1 | 0.54 | -0.88 | 0.0028 | 0.033 | no | down |
|  | 2 | 0.68 | -0.55 | 0.021 | 0.098 | no | down |
|  | 3 | 2.33 | 1.22 | 1.08×10^-5^ | 9.64×10^-5^ | yes | up |
|  | 4 | 1.57 | 0.65 | 0.084 | 0.17 | no | up |
| TRINITY_DN30358_c0_g1  (GSTU6) | 1 | 0.68 | -0.56 | 0.022 | 0.14 | no | down |
|  | 2 | 0.83 | -0.27 | 0.22 | 0.47 | no | down |
|  | 3 | 2.36 | 1.24 | 1.40×10^-8^ | 2.31×10^-7^ | yes | up |
|  | 4 | 1.46 | 0.55 | 0.12 | 0.22 | no | up |
| ^a^Compared group 1: LC-1701-C vs. LC-S-C; Compared group 2: LC-1704-C vs. LC-S-C; Compared group 3: LC-1701-T vs. LC-S-T; Compared group 4: LC-1704-T vs. LC-S-T.  ^b^Genes with an absolute value of log_2_fold-change (FC)≥1 and a *p*-value＜0.05 were considered differently expressed. | | | | | | | |

| **Table S12.** Cyhalofop-butyl toxicity on seven F_1_ groups of *Leptochloa chinensis*. | | |
| --- | --- | --- |
| Group | Slope (SE) | ED_50_ (95% CI)^a^ (g a.i. ha^-1^) |
| LC-S-F_1_ | 2.36 (0.13) | 9.8 (8.7-11.1) |
| LC-1701- F_1_ | 1.99 (0.21) | 138 (98-195) |
| LC-1704- F_1_ | 1.13 (0.19) | 565 (331-964) |
| LC-1701-ED_10_- F_1_ | 1.33 (0.20) | 166 (108-253) |
| LC-1701-ED_90_- F_1_ | 3.17 (0.74) | 194 (120-313) |
| LC-1704-ED_10_- F_1_ | 1.60 (0.13) | 440 (341-567) |
| LC-1704-ED_90_- F_1_ | 2.20 (0.15) | 522 (422-646) |
| ^a^95% confidence interval. | | |

| **Table S13.** Correlation analysis between constitutive expression of three candidate genes and cyhalofop-butyl resistance in certain F_1_ groups without treatment. | | | | |
| --- | --- | --- | --- | --- |
| Candidate gene | Group | Relative expression  (Mean±SE)^a^ | Related coefficient (R)^b^ | *P*-value^c^ |
| *CYP71Z18* | LC-S-F_1_ | 1.07±0.17 | 0.532 | 0.468 |
|  | LC-1704-F_1_ | 1.66±0.31 |  |  |
|  | LC-1704-ED_10_-F_1_ | 1.23±0.21 |  |  |
|  | LC-1704-ED_90_-F_1_ | 1.06±0.26 |  |  |
| *CYP71C1* | LC-S-F_1_ | 1.08±0.15 | 0.951 | 0.001* |
|  | LC-1701-F_1_ | 4.99±1.80 |  |  |
|  | LC-1704-F_1_ | 16.1±2.22 |  |  |
|  | LC-1701-ED_10_-F_1_ | 5.39±0.24 |  |  |
|  | LC-1701-ED_90_-F_1_ | 6.49±0.99 |  |  |
|  | LC-1704-ED_10_-F_1_ | 14.1±0.95 |  |  |
|  | LC-1704-ED_90_-F_1_ | 10.7±1.18 |  |  |
| *CYP81Q32* | LC-S-F_1_ | 1.02±0.08 | 0.147 | 0.754 |
|  | LC-1701-F_1_ | 1.56±0.07 |  |  |
|  | LC-1704-F_1_ | 2.30±0.17 |  |  |
|  | LC-1701-ED_10_-F_1_ | 1.92±0.15 |  |  |
|  | LC-1701-ED_90_-F_1_ | 1.34±0.13 |  |  |
|  | LC-1704-ED_10_-F_1_ | 0.87±0.03 |  |  |
|  | LC-1704-ED_90_-F_1_ | 1.01±0.13 |  |  |
| ^a^The relative expression levels are evaluated by the ratios of normalized expression of candidate genes in the corresponding group to that in the LC-S-F_1_ group using 2^-△△Ct^ method.  ^b^The related coefficients are calculated by Pearson correlation analysis.  ^c^An asterisk indicates a significant relationship between the gene expression level and cyhalofop-butyl resistance (*P*＜0.05). | | | | |

| **Table S14.** Correlation analysis between expression of eight candidate genes and cyhalofop-butyl resistance in certain F_1_ groups with herbicide treatment. | | | | |
| --- | --- | --- | --- | --- |
| Candidate gene | Group | Relative expression  (Mean±SE)^a^ | Related coefficient  (R)^b^ | *P*-value^c^ |
| *CYP71Z18* | LC-S-F_1_ | 1.04±0.11 | 0.379 | 0.621 |
|  | LC-1704-F_1_ | 2.46±0.29 |  |  |
|  | LC-1704-ED_10_-F_1_ | 0.72±0.15 |  |  |
|  | LC-1704-ED_90_-F_1_ | 0.96±0.15 |  |  |
| *CYP71C4* | LC-S-F_1_ | 1.06±0.15 | 0.998 | 0.002* |
|  | LC-1704-F_1_ | 2.37±0.30 |  |  |
|  | LC-1704-ED_10_-F_1_ | 2.00±0.19 |  |  |
|  | LC-1704-ED_90_-F_1_ | 2.22±0.22 |  |  |
| *CYP71C1* | LC-S-F_1_ | 1.07±0.15 | 0.989 | ＜0.001* |
|  | LC-1701-F_1_ | 5.44±0.96 |  |  |
|  | LC-1704-F_1_ | 51.6±3.45 |  |  |
|  | LC-1701-ED_10_-F_1_ | 8.35±1.20 |  |  |
|  | LC-1701-ED_90_-F_1_ | 17.7±1.81 |  |  |
|  | LC-1704-ED_10_-F_1_ | 38.7±7.63 |  |  |
|  | LC-1704-ED_90_-F_1_ | 44.9±2.80 |  |  |
| *CYP81Q32* | LC-S-F_1_ | 1.00±0.04 | -0.550 | 0.450 |
|  | LC-1704-F_1_ | 0.89±0.06 |  |  |
|  | LC-1704-ED_10_-F_1_ | 0.35±0.04 |  |  |
|  | LC-1704-ED_90_-F_1_ | 0.43±0.03 |  |  |
| *CYP76B6* | LC-S-F_1_ | 1.01±0.05 | -0.839 | 0.161 |
|  | LC-1704-F_1_ | 0.70±0.06 |  |  |
|  | LC-1704-ED_10_-F_1_ | 0.56±0.08 |  |  |
|  | LC-1704-ED_90_-F_1_ | 0.41±0.02 |  |  |

| **Table S14.** Correlation analysis between expression of eight candidate genes and cyhalofop-butyl resistance in certain F_1_ groups with herbicide treatment (continued). | | | | |
| --- | --- | --- | --- | --- |
| Candidate gene | Group | Relative expression  (Mean±SE)^a^ | Related coefficient  (R)^b^ | *P*-value^c^ |
| *GSTF11* | LC-S-F_1_ | 1.00±0.05 | 0.542 | 0.458 |
|  | LC-1701-F_1_ | 2.34±0.42 |  |  |
|  | LC-1701-ED_10_-F_1_ | 4.67±1.68 |  |  |
|  | LC-1701-ED_90_-F_1_ | 1.78±0.50 |  |  |
| *GSTF1* | LC-S-F_1_ | 1.01±0.07 | 0.996 | 0.004* |
|  | LC-1701-F_1_ | 4.24±0.73 |  |  |
|  | LC-1701-ED_10_-F_1_ | 5.48±0.78 |  |  |
|  | LC-1701-ED_90_-F_1_ | 5.94±0.59 |  |  |
| *GSTU6* | LC-S-F_1_ | 1.18±0.39 | 0.975 | 0.025* |
|  | LC-1701-F_1_ | 7.04±1.38 |  |  |
|  | LC-1701-ED_10_-F_1_ | 10.2±1.37 |  |  |
|  | LC-1701-ED_90_-F_1_ | 13.2±1.90 |  |  |
| ^a^The relative expression levels are evaluated by the ratios of normalized expression of candidate genes in the corresponding group to that in the LC-S-F_1_ group using 2^-△△Ct^ method.  ^b^The related coefficients are calculated by Pearson correlation analysis.  ^c^An asterisk indicates a significant relationship between the gene expression level and cyhalofop-butyl resistance (*P*＜0.05). | | | | |

**Figure S1. Alignment of the amino acid sequences of entire chloroplastic ACCase CT domain in *Leptochloa chinensis* and other gramineous weed species.** Different amino acids of the ACCase CT domain among these weeds are shaded blue. LC-S-1/2/3: Three types of ACCase CT domain in susceptible *Leptochloa chinensis*; EI: *Eleusine indica* (NCBI accession No. AHC53984.1); AM: *Alopecurus myosuroides* (NCBI accession No. CAC84161.1); EC: *Echinochloa crus-galli* (NCBI accession No. ADR32358.1).


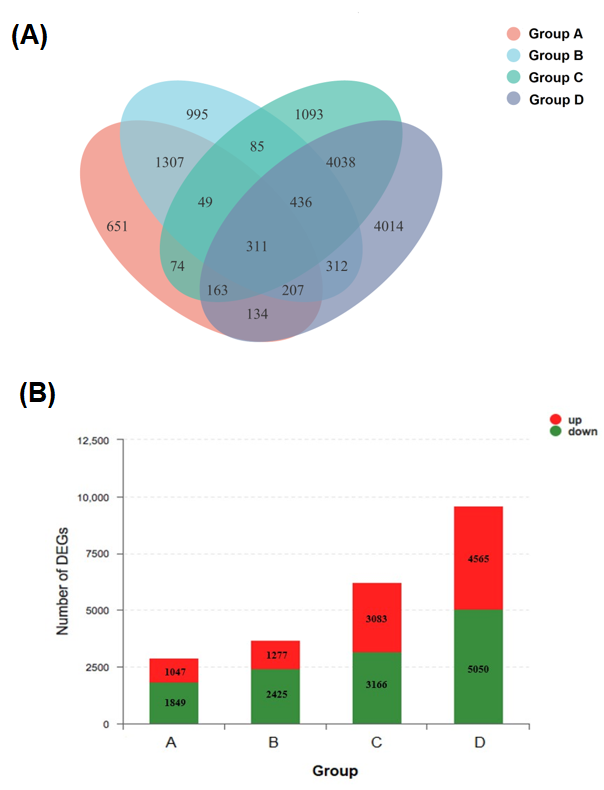


Figure S2. Venn diagram and numbers of DEGs in different *Leptochloa chinensis* groups. Group A: LC-1701-C vs. LC-S-C; Group B: LC-1704-C vs. LC-S-C; Group C: LC-1701-T vs. LC-S-T; Group D: LC-1704-T vs. LC-S-T. (A): A value of p < 0.05 was used as the threshold for the significance of DEGs. (B): Compared with the susceptible samples, the red and green columns that are labeled with numbers represent the amounts of up-regulated and down-regulated genes in the corresponding resistant samples, respectively.


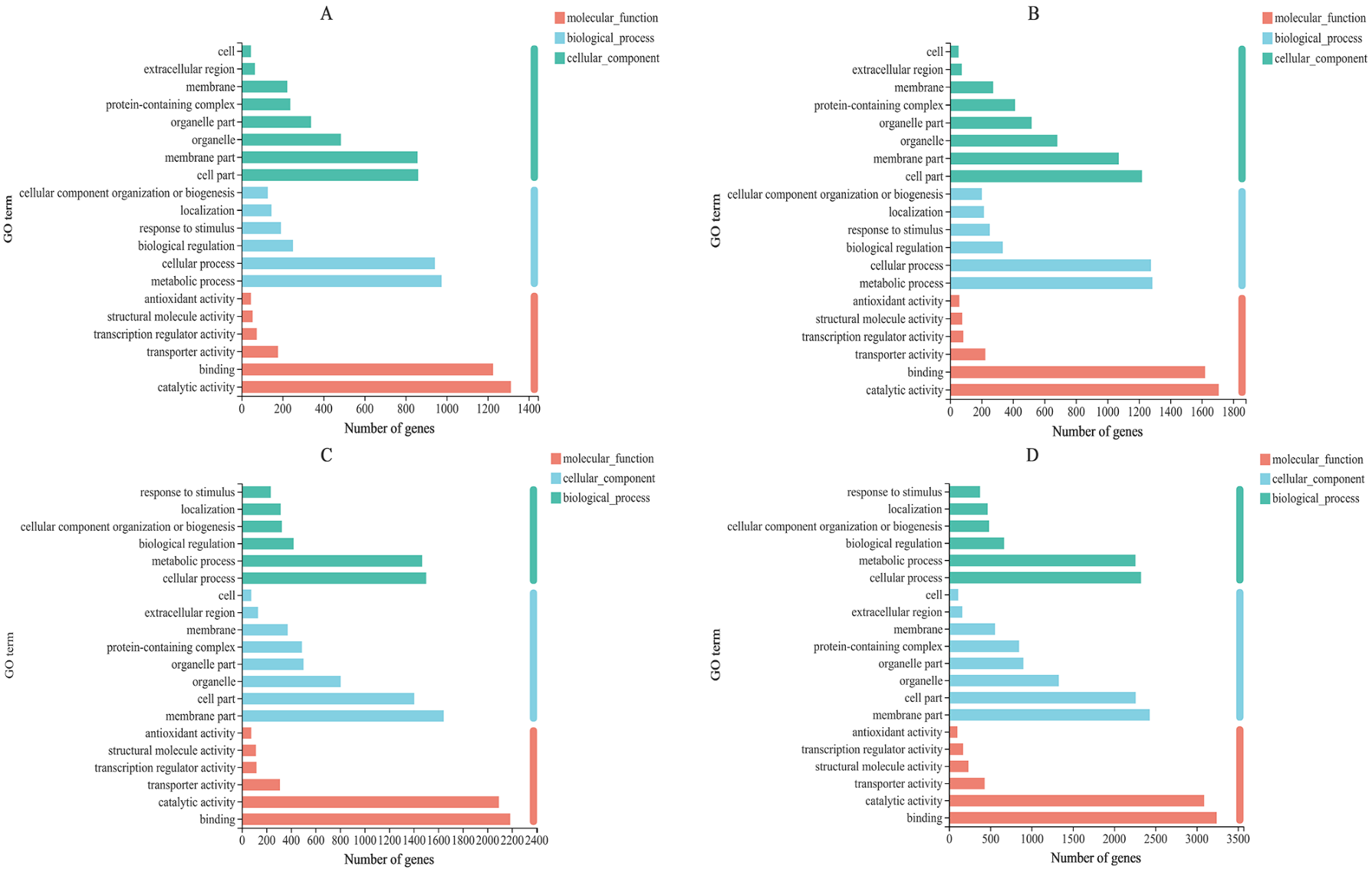


Figure S3. Gene ontology (GO) classifications of DEGs from four *Leptochloa chinensis* groups. A: LC-1701-C vs. LC-S-C; B: LC-1704-C vs. LC-S-C; C: LC-1701-T vs. LC-S-T; D: LC-1704-T vs. LC-S-T.
